# Need-Based Up-Regulation of Protein Levels in Response to Deletion of Their Duplicate Genes

**DOI:** 10.1371/journal.pbio.1000347

**Published:** 2010-03-30

**Authors:** Alexander DeLuna, Michael Springer, Marc W. Kirschner, Roy Kishony

**Affiliations:** 1 Department of Systems Biology, Harvard Medical School, Boston, Massachusetts, United States of America; 2 Laboratorio Nacional de Genómica para la Biodiversidad, CINVESTAV, Irapuato, Guanajuato, Mexico; 3 School of Engineering and Applied Sciences, Harvard University, Cambridge, Massachusetts, United States of America; University of Bath, United Kingdom

## Abstract

Many duplicate genes maintain functional overlap despite divergence over long evolutionary time scales. Deleting one member of a paralogous pair often has no phenotypic effect, unless its paralog is also deleted. It has been suggested that this functional compensation might be mediated by active up-regulation of expression of a gene in response to deletion of its paralog. However, it is not clear how prevalent such paralog responsiveness is, nor whether it is hardwired or dependent on feedback from environmental conditions. Here, we address these questions at the genomic scale using high-throughput flow cytometry of single-cell protein levels in differentially labeled cocultures of wild-type and paralog-knockout *Saccharomyces cerevisiae* strains. We find that only a modest fraction of proteins (22 out of 202) show significant up-regulation to deletion of their duplicate genes. However, these paralog-responsive proteins match almost exclusively duplicate pairs whose overlapping function is required for growth. Moreover, media conditions that add or remove requirements for the function of a duplicate gene pair specifically eliminate or create paralog responsiveness. Together, our results suggest that paralog responsiveness in yeast is need-based: it appears only in conditions in which the gene function is required. Physiologically, such need-based responsiveness could provide an adaptive mechanism for compensation of genetic, environmental, or stochastic perturbations in protein abundance.

## Abstract 2

Duplicated genes compensate for loss of one of the paralogs by up-regulating the remaining paralog only under growth conditions in which paralog activity is required for survival.

## Author Summary

Despite sequence divergence over long evolutionary times, many genes that have undergone duplication can still compensate for the loss of their duplicates. This compensation depends, not only on functional overlap between the paralogous genes, but also on overlap in their expression patterns. It has been proposed that compensation might therefore involve active up-regulation of a gene in response to deletion of its paralog. To test for such paralog responsiveness in the yeast *Saccharomyces cerevisiae*, we systematically measured changes in single-cell protein levels for approximately 200 duplicate genes in the presence or absence of their paralogs. Only a small fraction (∼11%) of proteins increased in level in response to deletion of their paralog, but this set matched almost exclusively the subset of paralogs whose overlapping function is required for viability. Further, when we examined yeast grown in different media, we found that genes had either gained or lost paralog responsiveness exactly according to their importance for growth in the tested conditions. Responsiveness, therefore, is need-based: it appears only in conditions in which the function of one or both paralogs is required. We propose that such need-based responsiveness of duplicate genes could play an important adaptive role, not just in the artificial event of paralog deletion, but also in the maintenance of functions that are compromised by natural genetic, environmental, or stochastic perturbations.

## Introduction

Gene duplication is a primary mechanism for the origin of new genes, providing raw material for functional innovation [1]–[8]. Small-scale duplication of individual genes as well as whole-genome duplication shape the genome of organisms from ciliates [9] and yeasts [10]–[12] to plants [13]–[15] and chordates [16],[17]. Following duplication, paralogous genes may assume different fates, including loss of one of the duplicates, divergence and functional differentiation, or maintenance of partially overlapping functions [7].

Although most paralogs are lost [18], some are retained. In the yeast *Saccharomyces cerevisiae*, genes that encode enzymes, transporters, and transcription factors have often survived in duplicate after a whole-genome duplication event that occurred 100 million years ago [7],[19],[20]. Furthermore, many surviving paralogs maintain overlapping functions despite divergence through long evolutionary time scales [21]–[24]. This functional overlap between duplicate genes manifests as synthetic aggravating interactions between paralogs; a double knockout of both duplicate genes shows a large phenotypic effect [21]–[24] despite the fact that each of the single knockouts shows a neutral or very weak phenotypic effect [21],[25].

In addition to functional overlap between the duplicates, the phenotypic buffering of an individual knockout requires expression of its paralogous gene. Analysis of transcriptional expression profiles has suggested the existence of “responsive backup circuits” that up-regulate a duplicate gene when its paralog is absent [26],[27]. Although several specific examples of gene dosage compensation between duplicate genes have been revealed in different organisms and biological processes [28]–[31], the genome-wide extent of such paralog-responsive backup circuits is unclear [32]. In principle, the ability of a gene to compensate for the absence of its paralog may be based on its basal protein expression level and not necessarily require its up-regulation.

By comparing single-cell levels of yeast proteins fused to the green fluorescent protein (GFP) in the wild-type and in the paralog-deleted background in *S. cerevisiae*, we systematically identified changes in protein levels for approximately 200 duplicate genes in response to deletion of their paralogs and revealed the environmental requirement for paralog responsiveness.

## Results

### High-Throughput Measurement of Differential Protein Levels in Wild-Type and Paralog-Deleted Strains

**Figure 1:**
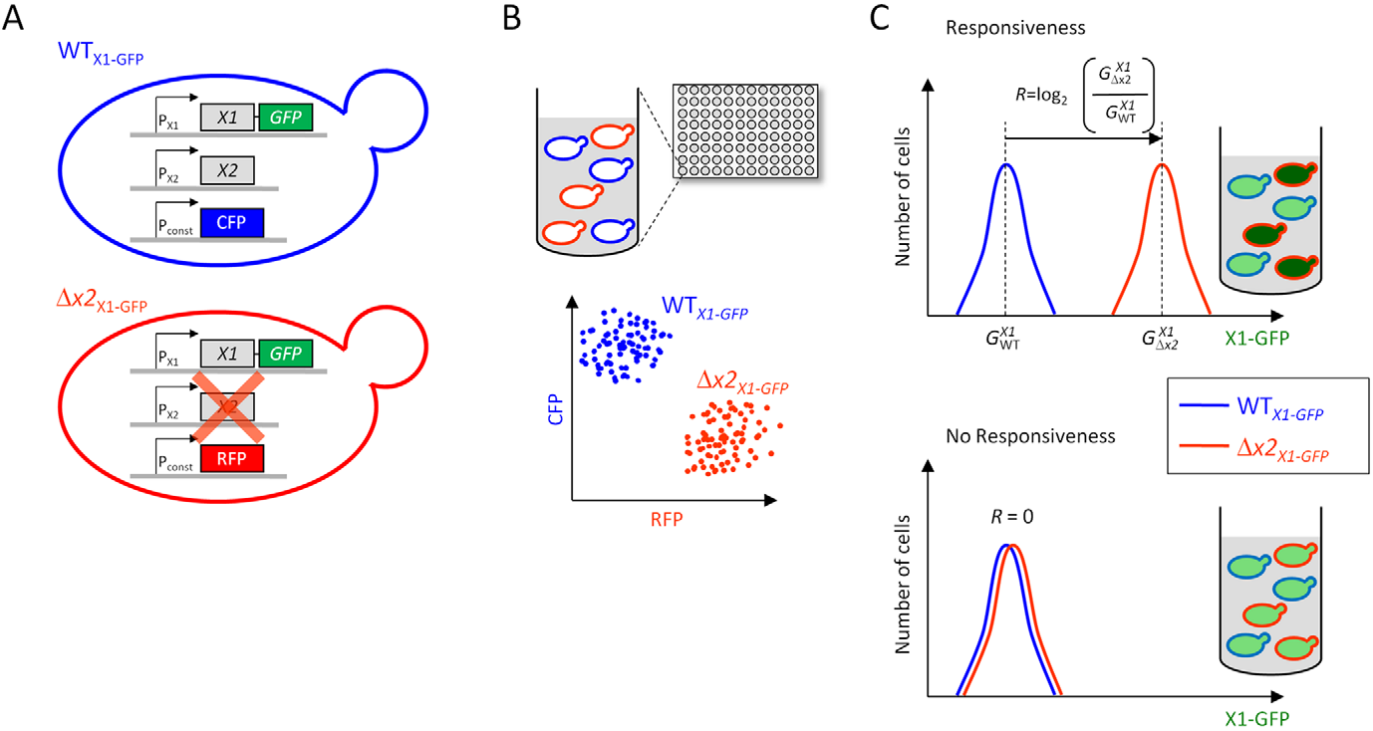
Systematic analysis of expression of proteins in response to deletion of their paralogs. (A) Pairs of haploid yeast strains were constructed in which a duplicate gene, *X1*, fused to GFP is expressed at its endogenous locus in either the wild-type background (WT*_X1-GFP_*) or in a background deleted for its paralog (Δ*x2_X1-GFP_*). These strains also constitutively expressed either cerulean or mCherry, respectively (CFP, RFP; dye swaps were also made). (B) For each gene *X1*, the matching strain pair WT*_X1-GFP_* and Δ*x2_X1-GFP_* were grown as cocultures in the same well of a 96-well plate. Three-color flow cytometry was used to distinguish wild-type versus *X2*-deleted cells. (C) Three-color flow cytometry was used to measure the distribution of X1-GFP expression for each of these cocultured strains. Responsive genes have a higher expression level when their paralog is deleted (top), whereas nonresponsive genes do not change their expression (bottom). Responsiveness (*R*) is defined as 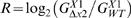, where 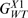 and 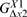 are the mean expression level of X1-GFP in the wild-type and in the Δ*x2* backgrounds (Materials and Methods).

To quantify the effect of deletion of a gene, *X2*, on the protein abundance of its paralog, *X1*, we used high-throughput flow cytometry to measure the level of X1-GFP fusion protein expressed at its endogenous locus [33],[34] in wild type and Δ*x2* haploid background strains (Figure 1). We constitutively expressed a marker fluorescent protein (cerulean [CFP] in the wild type strain and mCherry [RFP] in the Δ*x2* strain, or vice versa as a “dye swap” control), to provide a method for distinguishing mixed cells of the two strains. This allowed us to coculture the two strains, thereby ensuring that they were grown under identical environmental conditions, and to use flow cytometry to identify wild-type and knockout cells on a cell-by-cell basis while measuring each cell's GFP signal (Materials and Methods). From this data, we defined the paralog responsiveness, *R*, of *X1* as the log_2_ of the ratio of its mean expression level in the Δ*x2* background (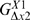) over the wild-type background (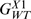), 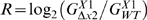.

We concentrated our analysis on 1,054 duplicate genes present in the yeast genome as two-member paralogous pairs [35]. Of this set of genes, 749 are available as protein fusions from the GFP-tagged yeast expression library [33], and for 92% of them, the corresponding paralog knockouts are present as viable strains in the yeast deletion collection [36]. Using two rounds of mating and haploid selection [37], we generated a total of 687 pairs of strains of GFP fusions in the paralog-deleted and wild-type backgrounds (Table S1). All ribosomal protein genes (54) were later removed from our collection to avoid potential complications due to aneuploidy, resulting in a total of 633 pairs of strains [38]. The libraries were constructed in quadruplicate—two replicates expressing CFP, and two replicates expressing mCherry (Materials and Methods; Figure S1).

We measured the GFP fluorescence of each protein fusion X1-GFP in mid-log phase in rich medium (YPD), in a 1∶1 coculture of wild-type and paralog-deletion strains (WT, Δ*x2*) in duplicate for each of the quadruplicate libraries (eight total replicates). After autofluorescence correction and spectral unmixing, GFP signal was detected for ∼50% of the X1-GFP protein fusions in both the wild-type and deletion backgrounds. Our results are restricted to the highest two thirds of these strains to ensure an accurate measurement of responsiveness, giving a total of 202 strains (Materials and Methods; Table S2).

To help remove nonspecific gene regulation of *X1* due to the physiological effect of *X2* deletion, we measured the effect of *X2* deletion on the expression of a housekeeping gene *RPL41B*. To this end, we generated a control library of Rpl41b-GFP fusions in each of the 633 deletion backgrounds discussed above, and in the wild-type background, respectively, tagged with CFP and RFP (and a “dye swap” control). Measuring the expression of Rpl41b-GFP in cocultures of each deletion strain and the wild type, we determined that 17 strains showed significant abnormalities in Rpl41b-GFP expression. Although these genes are interesting in their own right, we eliminated them from further analysis in this study (highlighted genes in Table S2).

### Some Genes Up-Regulate Expression in Response to Deletion of Their Paralogs

**Figure 2:**
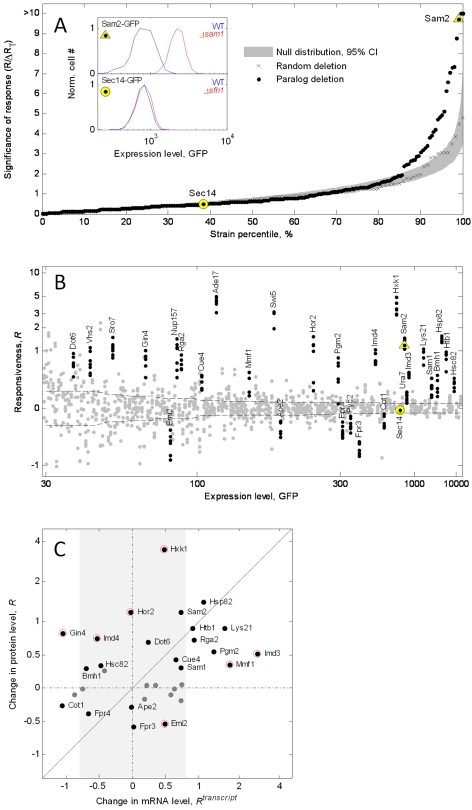
Certain duplicate genes show significant response to deletion of their paralogs. (A) Distribution of significance of responsiveness, measured as the level of significance *R* in units of standard error Δ*R_T_*, for the library of paralogous pairs (black dots), for a control library of random pairing (black crosses), and for the 95% confidence interval of the expected null distribution for responsiveness (gray band). Inset: histogram of the underlying flow cytometry data for one run of a highly significant responder (Sam2, yellow triangle) and one run of a nonsignificant responder (Sec14, yellow circle). The expression distribution is shown for the wild type (blue) and the background deleted for their paralogs (Δ*sam1* or Δ*sfh1*, red). The total error in responsiveness, Δ*R_T_*, is defined by (Δ*R_T_*)^2^ = (Δ*R_L_*)^2^ + (Δ*R_G_*)^2^. The local error Δ*R_L_* is defined as the standard deviation of all replicate experiments of a given gene (Figure S2); the global error Δ*R_G_* is defined as the average of Δ*R_L_* over a sliding window of expression levels (dashed line, see Material and Methods). (B) All the measurements for responsiveness, *R,* in six to eight replicate experiments for each gene (multiple dots in each column). Significantly responding genes are indicated (*R*/Δ*R_T_* >2, black dots). Genes are sorted by their wild-type expression level as indicated on the *x*-axis. (C) Correlation of protein-level responsiveness (*R*) with mRNA-level responsiveness (*R^transcript^*) of genes that respond (black-labeled dots) and do not respond (gray dots) at the protein level. The light-gray band is a significance cutoff for *R^transcript^* determined from replicate measurements (see Materials and Methods). Many of the genes that are up-regulated at the protein level also respond at the mRNA level, though some genes are significantly off the diagonal, suggesting posttranscriptional control (red circles, 95% confidence interval using the error from each individual measurement).

We found that only ∼15% (29) of the detectable duplicate genes are significantly up- or down-regulated in the paralog-deletion strain grown in rich medium (Figure 2A). Significance was determined using 95% confidence intervals derived by bootstrapping the set of measurements assuming no paralog responsiveness (*R* = 0) and using the measured noise in *R* (Figure 2A, gray band; Materials and Methods) with the actual distribution we observed. Noise in *R* was estimated from the variability in the replicate measurements of each gene (Figure 2B, Figure S2). We then constructed a control “random library” of X1-GFP fusions combined in random (nonparalogous) to the paralog-deletion backgrounds with a nonrelated deletion background. A total of 121 fusions in this set of strains had detectable GFP signal, and their responsiveness to the random deletion showed no significant deviation from the expected null distribution (Figure 2A, black crosses are inside the gray band). These controls indicate that the responsiveness we detected is specific to the deletion of the paralogous gene.

The majority (23 out of 29) of the paralog-responsive genes show positive responsiveness (*R*>0, up-regulation of gene in response to deletion of its paralog) and only few (six out of 29) showed negative responsiveness (Figure 2B). Following the backup hypothesis, we focus the rest of our analysis on the positively responding genes. We note though that negative responsiveness may also be an adaptive behavior, for example related to stochiometric regulation of protein complexes; indeed, we found that three out of the six negatively responding genes are known to interact physically with their paralogs (*FPR3*, *FPR4*, and *PYC2*) [39].

In the positively responding genes, we observed significant up-regulation from 1.13-fold to over 20-fold (median value 1.7-fold; Figure 2B; Table S2). For 78 GFP tagged proteins, we had data for both paralogs (39 pairs), and 11 genes responded positively within this set, including three pairs of mutually responding paralogs (*SAM1-SAM2*, *IMD3-IMD4*, and *HSP82-HSC82*; Figure S3). In the asymmetric cases—gene pairs in which one protein responds to deletion of its paralogous gene, but not vice versa—the responding protein can be either the high or the low expressed member of the pair (Figure S3).

Because previous backup circuit studies examined mRNA levels rather than protein levels, we asked whether the protein level responsiveness we observe occurs at the transcriptional or post-transcriptional level (Figure 2C). In analogy to the protein-level responsiveness *R*, we define the transcriptional responsiveness of a paralog *X1* as the log_2_ of the ratio of its mRNA expression levels in the Δ*x2* and the wild-type backgrounds, 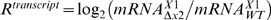. mRNA levels in the wild-type and paralog deleted backgrounds were measured by real-time PCR for most of the protein-responsive genes as well as for some nonresponsive controls (Materials and Methods; Table S3). The majority (25 out of 32) of the tested genes are consistent with transcription being the sole source of responsiveness (Figure 2C). Seven genes are interesting exceptions: *GIN4*, *IMD4*, *HOR2*, *HXK1*, *EMI2*, *MMF1*, and *IMD3*, which show significant difference between their mRNA and protein levels suggesting posttranscriptional control (Figure 2C, red circles). Strong translational up-regulation in the absence of transcriptional control has been previously observed for *HOR2* during osmotic stress [40],[41]. For *GIN4*, *IMD3*, and *MMF1*, there is significant opposing transcriptional and posttranscriptional regulation.

### Responsive Genes Appear Exclusively in Synthetically Interacting Paralogs

Are there any special features of paralog-responsive genes? We find that responsiveness is enriched in gene pairs that have similar expression profiles, regulatory motifs, and amino acid sequences (Figure S4). The functions of proteins that show responsiveness are very diverse. They include metabolic enzymes (e.g., Sam1, Ade17, Pgm2, Hxk1), cell-cycle proteins (Gin4, Pph22, Vhs2), Golgi proteins (Gga1, Sro7), and heat-shock proteins (Hsp82, Hsc82) (Figure 2B; Table S2). Amongst these, paralog-responsiveness is enriched in genes with metabolic function (*p* = 0.037, Fisher exact test). Further, paralog responsiveness is more likely to occur in genes expressed at high levels in the wild type (*p* = 0.01, Figure S5). Although high expression is correlated with metabolism [20],[42],[43], enrichment for high expression is significant even when accounting for a bias towards metabolic genes in the responsive set (Figure S5). This enrichment for highly expressed proteins raises the hypothesis that genes that contribute more to viability may show greater paralog responsiveness. Indeed, it has been suggested that responsiveness of functionally overlapping essential genes could provide a mechanism for compensation for perturbations in protein abundance [27].

**Figure 3:**
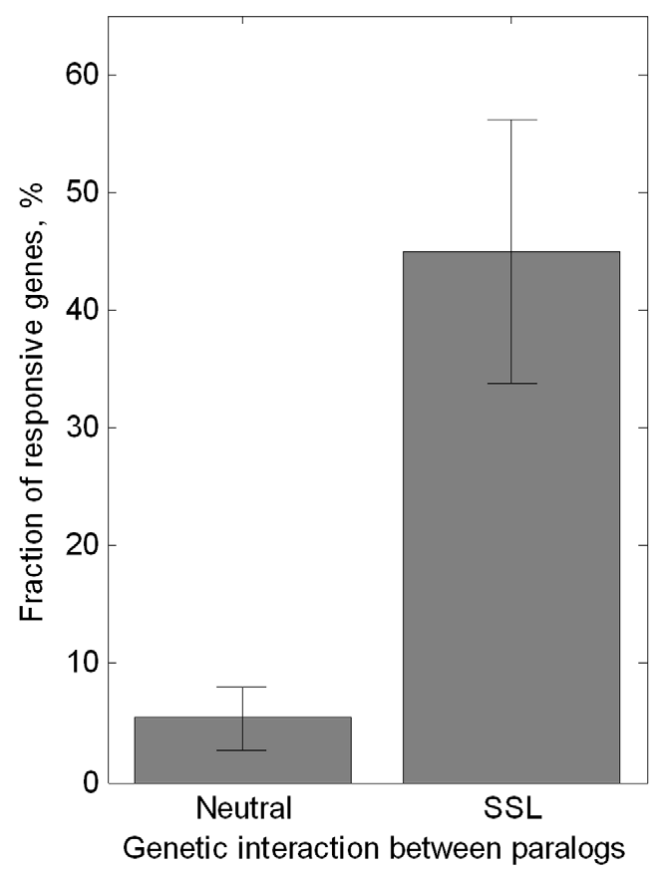
Paralog responsiveness in rich medium occurs almost exclusively in genes that are synthetic lethal or synthetic sick with their paralogs. Fraction of responding genes are shown for gene pairs with no genetic interaction (neutral, *n* = 37) and for synthetic lethal or sick interactions (SSL, *n* = 18). SSL interactions are defined as *ε*  = *f_x1x2_* − *f_x1_ f_x2_* <−0.2, where ε is the epistasis and *f_x1x2_*, *f_x1_*, and *f_x2_* are the fitness values for the double and single knockouts grown in rich YPD medium (fitness data taken from [22]). Error bars reflect binomial standard error of the mean. A similar trend is seen in minimal medium (Figure S6).

If responsiveness is related to viability, it should appear preferentially in paralogs that have overlapping essential functions in a given growth condition. Such paralogs with overlapping essential function should show synthetic interactions, i.e., deletion of both paralogs should have a much larger effect than expected from the effects of the single knock-outs. To test this idea, we compared our list of paralog-responsive genes in rich medium with a catalog of the phenotypes of single and double knockouts of duplicate genes characterized in the same conditions [22]. We categorized gene pairs into two classes: noninteracting (neutral) and synthetic sick/lethal interactions (SSL), according to whether the double-mutant growth rate is equal to or more severe than expected based on the growth rates of the two corresponding single mutants. We found that paralog responsiveness is strongly enriched in gene pairs with SSL interactions (Figure 3; *p* = 0.004, Fisher exact test), and very rarely observed in genes with neutral genetic interactions (Table S2; the only exceptions are *VHS2* and *CUE4*, which show marginally significant paralog responsiveness).

### Paralog Responsiveness Depends on Environmental Conditions

**Figure 4:**
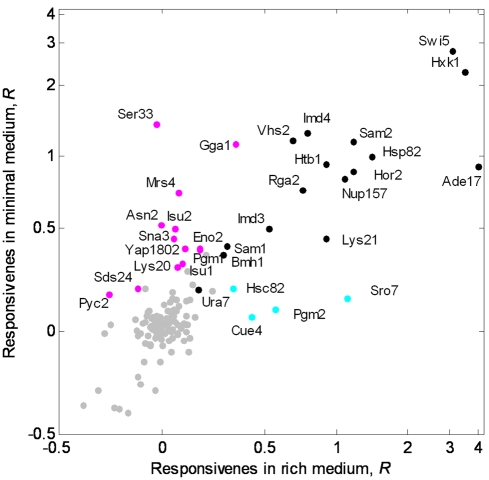
Responsiveness to paralog deletion shows condition specificity. Responsiveness, *R,* of each gene is shown in minimal versus rich medium. Genes are grouped into four classes: nonresponders (*n* = 106, gray), minimal-medium specific (*n* = 13, magenta), rich-medium specific (*n* = 4, cyan), and condition-unspecific response (*n* = 16, black). Gene names are indicated for all responding genes.

If responsiveness is enriched in gene pairs important for viability, one might expect to observe more paralog-responsive genes in a more metabolically challenging environment. To test this, we measured responsiveness in a nitrogen-poor minimal medium, using the entire set of paralog-deleted strains, and repeated the analysis of paralog responsiveness described for rich medium (Figure S6). We observed a new set of paralog-responsive genes specific to this medium (Figure 4, magenta dots). These genes include three functional classes: mitochondrial proteins with roles in iron regulation/function (Mrs4, Isu1, and Isu2); vesicular transport/regulation proteins (Yap1802, Gga1, Sna3, Sds24); and proteins involved in amino acid biosynthesis and glycosis (Ser33, Asn2, Pyc2, Pgm1, Eno2, and Lys20). Other genes are responsive in both conditions, or specific to rich medium, and the majority of genes do not respond in either condition (Figure 4, black, cyan, and gray dots).

We compared the paralog-responsive genes in minimal medium to quantitative data of SSL interactions between the paralogs under this condition [21]. Reinforcing the correlation observed in rich medium (Figure 3), we find that 50% of SSL gene pairs are paralog responsive, whereas none of the nonresponsive genes are SSL under these conditions (Figure S6; *p* = 0.001, Fisher exact test). This exclusiveness of paralog responsiveness to gene pairs with overlapping function critical for growth, together with the observation of amino acid biosynthetic genes showing paralog responsiveness specific to minimal media, indicate that responsiveness may be need-based, appearing only in conditions in which the gene's function is required.

### Paralog Responsiveness Is Specific to Conditions in Which the Gene Function Is Needed

To test the need-based responsiveness hypothesis more directly, we asked three questions: (1) Is the responsiveness of amino acid biosynthesis genes in minimal medium specific to environments that lack the amino acid? Likewise, (2) do genes that respond in both rich and nitrogen-poor conditions cease to respond in a condition that eliminates the need for their function? and finally, (3) do genes that do not respond in either condition respond in conditions in which their function becomes needed? We concentrated on several genes for which we could identify conditions that specifically generate or remove their functional need and measured their paralog-responsiveness under these conditions (see Text S1 for a detailed description of this set of genes).

**Figure 5:**
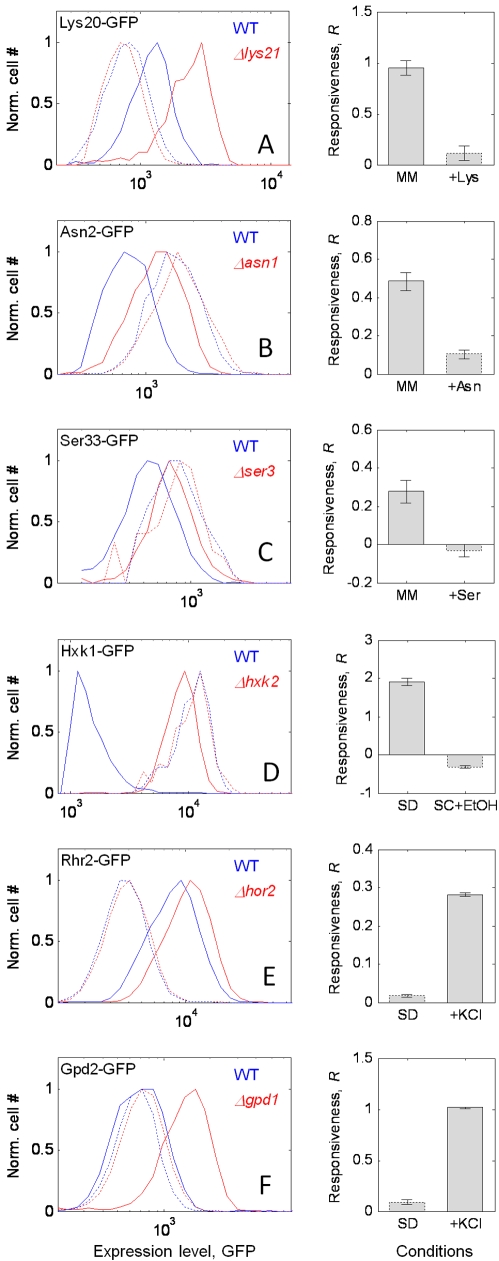
Responsiveness of a protein to deletion of its paralog is eliminated or created by removing or generating a need for its function. (A–F) Left: histogram of expression of a gene X1-GFP in the wild-type background (blue) and the paralog-deletion background (red). The protein fusions are (A) Lys20-GFP, (B) Asn2-GFP, (C) Ser33-GFP, (D) Hxk1-GFP, (E) Rhr2-GFP, and (F) Gpd2-GFP; histograms are shown for conditions in which the gene function is needed (solid lines) or unneeded (dashed lines). The total cell number in each sample was normalized (Norm. cell #). Right: responsiveness, *R*, of the focal gene in the needed (left bar) or unneeded (right bar) environments: MM, minimal medium; SD, synthetic complete dextrose medium; SC+EtOH, synthetic complete ethanol medium. Responsiveness and histograms reflect the median responsiveness value of three to 11 replicate experiments. Error bars indicate standard error of the mean.

For minimal-medium–specific responsive proteins, we concentrated on the amino acid biosynthesis enzymes Lys20, Asn2, and Ser33. We tested whether the responsiveness of these genes disappears when their respective amino acid is provided (Figure 5A–5C). Double mutants of *LYS20-LYS21*, *ASN1-ASN2*, or *SER3-SER33* are synthetic lethal in minimal medium, but viable if the relevant amino acid (lysine, asparagine, or serine) is added [44]–[47]. Thus, adding these amino acids removes the need for the corresponding gene pair. Indeed, we find that paralog responsiveness of Lys20-GFP, Asn2-GFP, and Ser33-GFP is specifically eliminated in the presence of lysine, asparagine, and serine, respectively (Figure 5A–5C). This loss of response upon complementation of the function appears in all three genes independently of their roles as the main or secondary isoform, and despite their different wild-type regulation by their cognate amino acid. Further, paralog responsiveness disappeared only upon the addition of the corresponding amino acid and not when any of the other amino acids was added (Figure S7; see legend for discussion of one exception). We conclude that paralog responsiveness of the amino acid biosynthesis genes is specific to an environment lacking the corresponding amino acid, namely to an environment in which the gene function is needed.

We then examined *HXK1* as an example of a gene that responded strongly in both rich and minimal media (Figure 4), and considered a new condition that would eliminate the need for its function. *HXK1* encodes hexokinase isoenzyme 1, which catalyzes the first irreversible step of glycolysis. This function will not be needed when cells are grown under a nonfermentable carbon source, such as ethanol. We find that the strong responsiveness of Hxk1-GFP seen in minimal glucose medium is completely abolished when cells are grown on ethanol as a source of carbon (Figure 5D); again, paralog responsiveness disappears when the gene's function is not needed.

Finally, we asked whether we could find conditions that would induce responsiveness in genes that do not respond in either rich or minimal medium (Figure 4, gray dots). We analyzed two nonresponding enzymes in glycerol biosynthesis pathway, Rhr2 and Gpd2, which are known to play a role in protection against osmotic stress. Although both Rhr2-GFP and Gpd2-GFP do not respond to deletion of their paralogs (*HOR2* and *GPD1*, respectively) in rich and synthetic complete media, they show strong paralog responsiveness in osmotic stress (0.5 M KCl; Figure 5E and 5F). Interestingly, this need-based response to paralog deletion occurs in *GPD2* despite the fact that it is not up-regulated by osmotic stress in the wild type (see [48] and Figure 5F, histograms). These results, therefore, reinforce our hypothesis that paralog responsiveness is specific to the conditions in which the gene function is needed.

## Discussion

Our quantitative protein-level measurements show that, in any given growth condition, responsiveness to paralog deletion is restricted to a small number of genes. Responsiveness occurs at both the transcriptional and posttranscriptional level. With almost no exceptions, such paralog responsiveness occurs only when the genes are synthetic lethal, namely, when they have an overlapping biochemical function that is critical for growth in the tested conditions. Removing or adding the need of a function, either by supplying its end product or by shifting to conditions in which its product is not required, specifically determines whether or not a given gene will respond to deletion of its paralog.

The mechanisms underlying need-based responsiveness are most likely complex. In principle, responsiveness of a gene to deletion of its paralog could reflect either a direct response to the absence of the paralogous protein (similar to supply control), or an indirect response to the absence of its function (similar to demand control [49]) (Figure S8A) [27]. A simple mathematical model of a metabolic pathway exemplify that indirect responsiveness should depend on the presence of the product of the pathway in the environment (Figure S8B and S8C; Text S2). Indeed, we found that for the amino acid biosynthetic genes, the addition of the amino acid end product eliminates paralog responsiveness (Figure 5A–5C), suggesting that responsiveness is not due to the absence of the paralogous protein but rather to the absence of its function. Such paralog responsiveness may therefore reflect a simple end-product regulation of genes. This supports the demand strategies previous identified in glycolysis [49]–[52]. Indeed, feedback regulation often occurs in the first committed step of a pathway, and these metabolic branching points are known to be enriched for duplicated genes [53],[54].

This logical argument is based on the notion that addition of the end product of a pathway supplements its biosynthetic function. The argument, therefore, does not apply to conditions that instead of supplying the end product simply remove the need of the function. For example, yeast cells need to accumulate glycerol only in osmotic stress; removing the osmotic stress relieves the need for the glycerol biosynthetic pathway not by externally supplying its end product, glycerol, but rather by generating conditions in which this end product is not needed. This is in contrast to the case of the amino acid biosynthetic genes; we therefore cannot conclude from our data that the mechanism underlying responsiveness of Hxk1, Rhr2, and Gpd2 is indirect. Indeed, the responsiveness of Hxk1 may be mediated by direct regulation of its paralog; nuclear Hxk2 is involved in repression of *HXK1* and expression of its own gene, *HXK2* [55],[56]. In agreement with these observations, we find that either the absence of glucose or the absence of *HXK2* results in Hxk1 up-regulation (Figure 5D). These differences in the underlying mechanisms of responsiveness underscore the breadth of its functional roles and suggest that in some cases, responsiveness to paralog deletion could even depend on the presence of other (nonparalogous) genes [57].

Genetic redundancy is a salient feature of living organisms. It has long been discussed under what circumstances genetic redundancy is evolutionary stable [58]–[60] and how redundancy can contribute to genetic robustness [61]–[63]. Interestingly, we uncovered a set of genes that are not up-regulated under a specific condition unless their paralogs are deleted. This and other cases of need-based responsiveness of genes to the absence of their paralogs could play an adaptive role in the compensation of functions that are compromised by genetic, environmental, or stochastic perturbations.

## Materials and Methods

### Strains and Media

Deletion strains were from the yeast deletion collection [36], *xxx*Δ*::KANMX4* in the S288C derivative BY4741 background (*MAT*a *his3*Δ*1 leu2*Δ*0 ura3*Δ*0 met15*Δ*0*). GFP protein fusions were obtained from the GFP library[33], XXX-GFP (S65T)::*SpHIS5MX6* in the same BY4741 background. Fluorescent starter strains Y8205-RFP and Y8205-CFP were generated by direct PCR-based gene replacement of the neutral *HO* locus with the pFA6a cassettes mCherry-*NATMX4* (RFP) and yECerulean-*NATMX4* (CFP), respectively, in the Y8205 strain (*MAT*α *can1Δ::STE2pr-SpHIS5 lyp1*Δ::*STE3pr-LEU2 his3*Δ*1 leu2*Δ*0 ura3*Δ*0 met15*Δ*0*) [37]; strong constitutive expression of fluorescent proteins is driven by the *TDH3* promoter.

The following growth media were used: (1) rich medium: yeast extract peptone dextrose (YPD); (2) minimal nitrogen-poor medium (MM): yeast nitrogen base without amino acids and ammonium sulfate with 2% glucose, 0.2% proline as a nitrogen source, and supplemental methionine (25 mg/l); (3) minimal nitrogen-poor medium with 1 mg/l lysine (MM+Lys), 1 mg/l asparagine (MM+Arg), or 1 mg/l serine (MM+Ser); (4) SD: synthetic complete medium with 2% glucose; (5) SC-EtOH: synthetic complete medium with 2% ethanol; or (6) SC+KCl: synthetic complete with 2% glucose and 0.5 M KCl.

All strains in this study are prototrophic except for methionine production. To confirm that supplied methionine levels were not having a major effect on our results, we examined responsive under two different methionine concentrations 25 mg/l (the amount used in the standard growth medium for logarithmic growth [64]) and 100 mg/l (the amount needed for maximal yield of cells at saturation [65]). Our results were largely unaltered by changing methionine levels (Figure S9).

### Generation of Yeast Libraries

Arrays of GFP-tagged proteins in wild-type and knockout backgrounds were generated by two rounds of synthetic genetic array methodology (SGA) [37]. Briefly, the RFP-tagged SGA starter strains were mated to an array of 687 deletion strains, Δ*x2*. This mating step was followed by diploid selection, sporulation, and three rounds of haploid selection (−LEU for alpha mating type, +G418 for knockout, and +clonNAT for fluorescence marker selection). In a second SGA round, the resulting arrays were crossed to their paralogous corresponding strains *X1-GFP* from the GFP library [33], and the diploids were selected (−LEU −HIS +G418 +clonNAT selection). To obtain the same X1-GFP fusion in a wild-type background with a different color tags, the CFP-tagged starter strain was mated to a strain with a neutral *KANMX4* insertion at the *his3*Δ1 locus. Dye swaps (deletion in CFP and wild-type in RFP) were also generated as described above. The libraries were constructed in quadruplicate—two replicates of the two dye swaps. Colony arrays were transferred manually with a 384-head pin tool (V&P Scientific, VP384F); antibiotic concentrations used for selection were 200 µg/ml G418 (Invitrogen), 100 µg/ml clonNAT (Werner BioAgents). A schematic of the entire strain generation procedure is shown in Figure S1.

Quality control testing of the strain arrays included: (1) fluorescence intensity of the entire library by flow cytometry and correlation with data from the literature [34]; (2) verification of GFP subcellular localization by microscopy of 50 random strains based on the reported protein localization [33]; and (3) PCR verification of the insertion site for one eighth of the rearrayed deletion library. These tests indicated that one of the four replicates was systematically inconsistent for one half of the arrays (*X1-GFP* not matching its corresponding Δ*x2*). These strains were eliminated for further analysis, leaving three replicates instead of four for approximately one half of the data. Ninety percent to 95% of the remaining strains were confirmed as correct for GFP fluorescence intensity and localization, and for deletion site.

Finally, two control libraries were generated following the SGA steps described above. The first control library contained a constant GFP fusion of the ribosomal protein *RPL41B* in either a wild-type background or one of the 687 deletions described above. A second control library of 364 GFP-fusions with random (nonparalogous) deletion backgrounds was constructed by crossing an array of GFP fusion strains to the inverted corresponding array of deletion collection strains. As for the main *X1-GFP* Δ*x2* library, two replicates of the two dye swaps were generated for these control libraries.

### Preparation of Cocultures

Each library was grown individually to saturation in 96-well plate format. Medium (600 µl) was dispensed with a MicroFill Microplate Dispenser (BioTek) onto 1.0-ml polypropylene plates (Nunc 260251), and cultures were incubated in a Multitron Infors platform shaker at 30°C with shaking at 999 rpm. Each experimental run involves coculturing two libraries; one constitutively expressing CFP and the other constitutively expressing RFP. The two libraries were mixed in one 96-well plate by combining equal volumes of liquid from the saturated library plates described above. A 96-pin tool (V&P Scientific, VP 407) was then used to inoculate a fresh plate in the medium of interest. Strains were then grown to mid-log phase (∼10 h in YPD or ∼14 h in MM). To analyze the libraries, cells were first transferred into 100 µl of TE (10 mM Tris and 1 mM EDTA [pH 8]), by two rounds of centrifugation at 3,000 *g* for 3 min, followed by liquid removal and resuspension in 600 µl of TE. Each pair of X1-GFP *X2* and X1-GFP Δ*x2* was measured six to eight times (two replicates of three to four independently constructed strains).

### Flow Cytometry: Instrumentation, Acquisition, and Data Analysis

A flow cytometer with a high-throughput autosampler (LSRII with a HTS, Becton Dickinson) was used to record fluorescence from GFP, CFP, and RFP fluorophores. GFP was excited with a 488-nm laser, and fluorescence was collected through a 525/50 band-pass and 550LP emission filter. CFP was excited with a 405-nM laser, and fluorescence was collected through a 450/50 band-pass filter and a 505LP emission filter. RFP was excited with a 593.5-nm laser, and fluorescence was collected through a 630/20 band-pass and a 640LP emission filter. Cells were measured in high-throughput mode at a flow rate of 0.5 µl/s for 8 s.

Data analysis was performed largely as described by Newman et al. [34] with the exception of using a trimmed mean and a less stringent size cutoff. Custom Perl and Matlab scripts using FCSread.m (Robert Hanson, available at Matlab central) were written to import the FCS raw data (*G_raw_*, GFP; *C_raw_*, CFP; *R_raw_*, RFP). For each well, analysis followed the following steps: (1) Remove cell debris and aggregates based on the forward and side scatter (an approximation of cell size). (2) Correct for crosstalk between fluorophores: *C* = *C_raw_* − *G_raw_* /10. (3) Classify the cells into RFP expressing (if *R_raw_* /*C* >20) or CFP expressing (if *C*/*R_raw_* >20), and record the GFP level 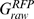 and 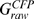 from these two population, respectively. This classification eliminates dead cells (no fluorescence in either channel) and doublets (fluorescence in both channels; appeared at rate of less than 1%). (4) Eliminate the 10% outlier values of 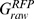 and 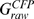 (5% strongest and 5% weakest). (5) Calculate the mean (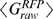, 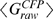) and standard deviation (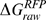, 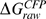) of the GFP fluorescence of each population. (6) Correct for autofluorescence and crosstalk: 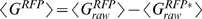 and 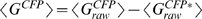, where 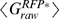 and 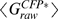 are the mean GFP fluorescence of 40 control strains expressing only the RFP or CFP, but not GFP.

Any strain that did not have GFP fluorescence in both the wild-type and deletion strains greater than 50% above the background fluorescence or a GFP fluorescence greater than twice the background in either of the strains was eliminated. This eliminated ∼66% of the strains. This is a more stringent cutoff than previous metrics, which solely tried to determine the number of strains above background and were able to detect 50% of all strains [34].

### Paralog-Responsiveness Metric And Error Analysis

The responsiveness was calculated as *R* = log_2_(*G^RFP^*/*G^CFP^*), for mutant RFP and wild-type CFP, or *R* = log_2_(*G^CFP^*/*G^RFP^*) for the reverse “dye swap.” Multiple lines of evidence support the use of GFP fusion proteins to accurately reflect responsiveness of the endogenous proteins. First, based on tagging of essential and nonessential proteins, most GFP-fusions are believed to generate functional proteins [33],[34]: i.e., genes missing from the GFP and TAP fusion collections are not enriched for essential genes. Second, protein levels determined by mass spectrometry give similar protein levels as those determined by flow cytometry of GFP fusions [66]. Third, our method is ratiometric. Even if the GFP fusion affected the protein levels (e.g., through stability or translatability), our method would only erroneously detect responsiveness if such presumed artificial effect of the GFP fusion was altered by the presence or absence of the paralog of the gene. Finally, independent measurements of responsiveness of tagged and untagged proteins for several genes by Western blot give very similar results to the GFP fluorescence measurements (Figure S10).

The median and standard deviation of the responsiveness metric was calculated from the six to eight replicates of measurements of responsiveness of each gene. For each strain, we calculated the “local error” Δ*R_L_* as the standard deviation of *R* of that strain over its six to eight replicate measurements. As seen in Figure S2A, this value is influenced by the total fluorescence of the strain. Due to the inaccuracy of calculating the standard deviation with six to eight measurements, we also calculated a global error, Δ*R_G_*, which is a moving-window median of the local error of 41 adjacent measurements sorted by total fluorescence (Figure S2A, dashed line). The total error that we then used for statistics was Δ*R_T_*, defined by (Δ*R_T_*)^2^ = (Δ*R_L_*)^2^ + (Δ*R_G_*)^2^. The replicate measurements within the same dye-swap had much smaller variance compared to the difference between the dye-swaps. Therefore, we used 2 as the effective number of independent measurements and calculated the standard deviation of the mean as Δ*R_T_*/√2. A null hypothesis was then generated by simulating the experiment (global and local error for each strain) by randomly sampling a normalized Gaussian distribution. This was repeated 100,000 times and the 95% confidence interval determined from this simulated dataset.

### Reverse Transcriptase Real-Time PCR Analysis

We measured mRNA levels of our GFP fusion proteins using quantitative PCR (qPCR). Wild-type *X1-GFP* and Δ*x2 X1-GFP* strains were separately grown in 30 ml of YPD and harvested at mid-log phase after 10 h of growth. Total RNA was extracted and cDNA was obtained from each sample using reverse transcriptase (Superscript III RT, Invitrogen), which was used as a template for real-time PCR using primer pairs to amplify GFP and a control gene *ACT1* from each sample. Because each gene in our study was GFP tagged, a universal set of GFP primers could be used. To normalize for variations in mRNA extraction, the *X1-GFP* mRNA level was defined relative to the *ACT1* level, 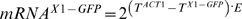, where *E* is the PCR efficiency and *T* is the product detection time in number of qPCR cycles. Paralog responsiveness at the mRNA level was then calculated as 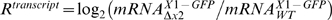. Table S3 contains the qPCR data. Expression levels were obtained from at least three technical qPCR replicates. To obtain an estimate for the experimental variation in our measurement, *R^transcript^* was measured in duplicate for Cot1, Hxk1, and Sam1, and in triplicate for Sam2 (see Table S3). The standard deviation of log_2_(mRNA) was 0.25, yielding standard deviation of 0.4 in *R^transcript^*. We used a significance cutoff of two standard deviation (95% confidence interval), or 0.8, for *R^transcript^* (gray shaded area in Figure 2C).

### Western Blot

Anti-yeast hexokinase antibodies (ABCAM ab34588) were used to detect Hxk1 and Hxk2; Lys20 and Lys21 were detected with Lys 20p + 21p antibody (ABCAM ab4574). Lys20 and Lys21 can be separated by electrophoretic mobility. We could not electrophoretically separate Hxk1 and Hxk2. To monitor the untagged version of Hxk1, we therefore monitored its level in the absence or presence of Hxk2-GFP. Hxk2-GFP is electrophoretically separable from Hxk1 and hence does not interfere with the measurement of the untagged Hxk1. We similarly examined Hxk2 in an Hxk1-GFP background. Samples were lysed in boiling 2× Laemlli buffer in the presence of a protease inhibitor cocktail (PMSF PLUS Roche #11836153001). Samples were run on precast NuPage (NP0321BOX) gels and transferred to nitrocellulose membranes. The Odyssey protocol was followed. Goat anti-mouse 680 (Alexa Fluor A-21057, 1∶5,000) and goat anti-rabbit 680 (Alexa Fluor A-21076, 1∶5,000) secondary antibodies were used. The fluorescence was quantified by Odyssey system (Li-COR). All measurements were made in duplicate or triplicate. The linearity of each antibody was confirmed by titrating both the primary antibody concentration and the substrate concentration. The working dilutions were 1∶2,000 and 1∶500 for the Hxk1/2 and Lys20/21 antibodies, respectively. The hexokinase antibody also reacted with a nonspecific band that was unaffected by medium and genetic background. Hxk1/2 antibody was used to detect this background band (C, control) for quantification in Figure S10. We also used a CEP3 and ACT1 antibody to control for loading, but the standard deviation of all our replicate measurements was lowest when normalized against the background band detected with the Hxk1/2 antibody.

## Supporting Information

Figure S1 **Schematic of library construction.** Yeast strain libraries were generated as described in Materials and Methods. In a first SGA round, libraries of mCherry- or Cerulean-tagged deletion or wild-type strains were generated. In a second SGA round, these arrays were combined with strains from the GFP library, generating the X1-GFP Δ*x2* and X1-GFP *X2* libraries. Black solid cross/arrows denote SGA mating, sporulation, and selection steps.
(0.49 MB TIF)

Figure S2 **Analysis of measurement error of paralog-responsiveness.** (A) Responsiveness of each gene was measured in multiple replicates representing four independently constructed strains (two of each CFP/RFP dye-swap variant), assayed in two independent replicates of the measurement procedure on different days (eight replicates total). Responsiveness *R* of each gene X1 was evaluated independently for each of its eight replicates as *R* = log_2_(*G_Δx2_*/*G_WT_*), where *G_WT_*, *G_Δx2_* are the 5% truncated mean expression level of X1-GFP in the wild-type and in the Δ*x2* backgrounds, respectively. For each gene, the standard deviation of *R* in all its replicate measurements defines its “local error” Δ*R_L_* (grey dots). The global error Δ*R_G_* is then defined as the average of Δ*R_L_* over a sliding window of expression levels (dashed line, Materials and Methods). The total error for each gene Δ*R_T_* is defined by (Δ*R_T_*)^2^ = (Δ*R_L_*)^2^ + (Δ*R_G_*)^2^. (B) Responsiveness of each gene is plotted as a function of its wild-type expression level. Vertical error bars represent Δ*R_L_*. Dashed line indicates 2Δ*R_G_*. Significant genes have total error *R*/Δ*R_T_* >2 (colored names).
(0.30 MB TIF)

Figure S3 **Responsiveness can be asymmetric and a property of either the low or higher or high expressed protein.** Wild-type protein expression levels as determined by Western blot of TAP-tagged proteins [67] are compared for each paralogous pair. Red dots represent pairs where both paralogs are responsive, green dot where one of the two paralogs is responsive, and grey dots where neither of the paralogs are responsive. When one pair is responsive, the responsive protein expression level is plotted on the *x*-axis. As responsiveness is limited to cases where we measured GFP expression, a subset of the grey dots could be green (asymmetrically responsive gene pairs) or red (symmetrically responsive), and a subset of the green dots could be red (symmetrically responsive).
(0.21 MB TIF)

Figure S4 **Responsiveness correlates with conservation of expression profiles, regulatory motifs, amino acid sequence, and fitness cost of paralog deletion.** (A–F) Fraction of responsive genes as a function of (A) mean expression similarity, (B) partial coregulation, (C) fraction of common *cis*-regulatory motifs, (D) Ks rate of amino acid divergence, (E) number of shared protein domains, and (F) fitness cost upon deletion of the *X2* paralog, as downloaded from Kafri et al. (http://longitude.weizmann.ac.il/BackUpCircuits/) [26]. The relevant dataset was ranked and split into three groups of equal data size. Error bars indicate standard error of the mean.
(0.48 MB TIF)

Figure S5 **Paralog-responsiveness is enriched in highly expressed proteins.** Fraction of responsive genes are shown for protein fusions with low (*G_WT_* >500) and high (*G_WT_* >500) expression levels, separated into metabolic (black) and nonmetabolic (grey) genes. Error bars represent binomial standard error of the mean. Paralog responsiveness is enriched in highly expressed proteins (*p* = 0.01) and slightly in metabolism (*p* = 0.037). Enrichment in highly expressed proteins is significant even when accounting for enrichment in metabolism and for the correlation of metabolism with high expression (*p* = 0.007, logit regression).
(0.11 MB TIF)

Figure S6 **Paralog responsiveness in minimal media is strongly correlated with synthetic sick and lethal interactions.** (A) Shown are all the measurements for paralog responsiveness, *R,* in minimal media, including replicate experiments for each gene (multiple dots in each column). Significantly responding genes are indicated (*R*/Δ*R_T_* >2, black dots). Genes are organized by their wild-type expression level as indicated on the *x* axis (see Figure 2B, for the equivalent presentation of responsiveness in rich medium). (B) Fraction of paralog-responding genes in minimal media are shown for gene pairs with no genetic interaction (neutral, *n* = 27) and for synthetic lethal or sick interactions (SSL, *n* = 16) in these conditions. SSL interactions are defined as *ε*  = *f_x1x2_* − *f_x1_ f_x2_* <−0.2, where ε is the epistasis and *f_x1x2_*, *f_x1_*, and *f_x2_* are the fitness values for the double and single knockouts grown in minimal medium (fitness data taken from DeLuna et al. [21]). Error bars reflect binomial standard error of the mean. All paralog-responsive genes are also synthetic lethal or synthetic sick with its paralog.
(0.43 MB TIF)

Figure S7 **Paralog responsiveness is specific to the conditions in which the gene function is needed.** (A–E) Responsiveness, *R*, of the focal gene in the needed (light-gray bars) or unneeded (dark-gray bars) environment: MM, minimal medium; SD, synthetic complete dextrose medium; SC+EtOH, synthetic complete ethanol medium, +Ser, minimal medium plus serine; +Lys, minimal medium plus lysine; and +Asn, minimal medium plus asparagine. The protein fusions are (A) Lys20-GFP, (B) Asn2-GFP, (C) Ser33-GFP, (D) Rhr2-GFP, and (E) Gpd2-GFP. Responsiveness of these genes is greatly reduced when cells are grown in conditions in which the genes are not needed. An exception is Asn2, which stops responding not only in the presence of asparagine, but also in the presence of lysine. *R* reflects the median responsiveness value of three to 11 replicate experiments. Error bars indicate standard error of the mean.
(0.30 MB TIF)

Figure S8 **A model for direct and indirect paralog responsiveness.** (A) A simple metabolic pathway showing enzymatic reactions (grey arrows) between metabolites (black circles). A gene *X1* (tagged with GFP) may respond to deletion of its paralog *X2* by two conceptual ways: (1) directly, in response to the absence of the paralogous protein (black solid inhibitory line), or (2) indirectly, in response to the absence of the function of the gene, for example through inhibition by the pathway end product (blue dashed inhibitory line). Mathematical models for gene expression in these two schemes were created (Text S2). (B) In an environment with a fixed amount of the end product, direct and indirect regulation of *X1* in response to change in concentration of *X2* are almost indistinguishable. (C) Responsiveness of *X1* to deletion of *X2* (*X2* = 0) in the two models can be distinguished by supplying the pathway product.
(0.29 MB TIF)

Figure S9 **Methionine concentration has a minimal effect on the measurement of responsiveness.** Responsiveness was measured in duplicate at two methionine concentrations, 25 mg/l and 100 mg/l, for one fourth of the library. The difference in responsiveness between these two environments, Δ*R_M_* (*R* for growth in 100 mg/l methionine minus *R* for growth in 25 mg/l methionine) is plotted as a function of average log_2_ expression of the 25 mg/l methionine-grown strain. Local and global errors are indicated (*R_L_*, error bars; *R_G_*, dashed line; Materials and Methods). Gray dots do not change significantly between conditions; five proteins Isu2, Sds23, Sso2, and Pyc1 have significant changes in responsive between the conditions.
(0.15 MB TIF)

Figure S10 **Western blots of untagged proteins confirm responsiveness of GFP-fusion proteins.** (A–D) Hxk1 (H1), Hxk2 (H2), Hxk1-GFP (H1G), Hxk2-GFP (H2G), Lys20 (L20), Lys21 (L21), and a control protein (Act1, Lys20/21, or HXK1,2 [C, control]; see Materials and Methods) were detected by quantitative Western blot. The genotype of each strain used is listed beneath each lane (STD, protein standard; G, GFP fusion; +, wild-type untagged protein; –, deletion). Titration triangles indicate a 2-fold dilution of the sample. Lys20 and Lys21 could be resolved on a SDS-PAGE gel, but Hxk1 and Hxk2 could not. To resolve Hxk1 and Hxk2, each was GFP tagged to alter its mobility from the untagged protein being queried. Samples were grown in (A and C) YPD, (B) SC + 2% EtOH, and (C) minimal medium. (D) All the measurements were quantitated with a fluorescent secondary using the Odyssey software (Materials and Methods), and the responsiveness *R* was calculated as the ratio of its level in the mutant and the wild type corrected for the loading controls (specific formula indicated below each bar). The error bars represent the standard deviation of the replicate measurements. Responsiveness is not significantly altered by tagging or method of quantitation (Western versus flow cytometry).
(1.72 MB TIF)

Table S1 **Library of yeast strains generated for this work.**
(0.23 MB XLS)

Table S2 **Complete dataset.**
(0.24 MB XLS)

Table S3 **Complete real-time qPCR dataset.**
(0.04 MB XLS)

Text S1 **Regulatory mechanisms underlying responsiveness under different environmental conditions.**
(0.05 MB DOC)

Text S2 **Model for direct and indirect responsiveness.**
(0.10 MB DOC)

## Abbreviations

qPCR: quantitative PCR
SSL: synthetic sick/lethal interaction
