# Supplementary figures and images for: Need-Based Up-Regulation of Protein Levels in Response to Deletion of Their Duplicate Genes
Source: PLoS Biol. 2010 Mar 30;8(3):e1000347. doi: 10.1371/journal.pbio.1000347 (PMC2846854; doi:10.1371/journal.pbio.1000347)

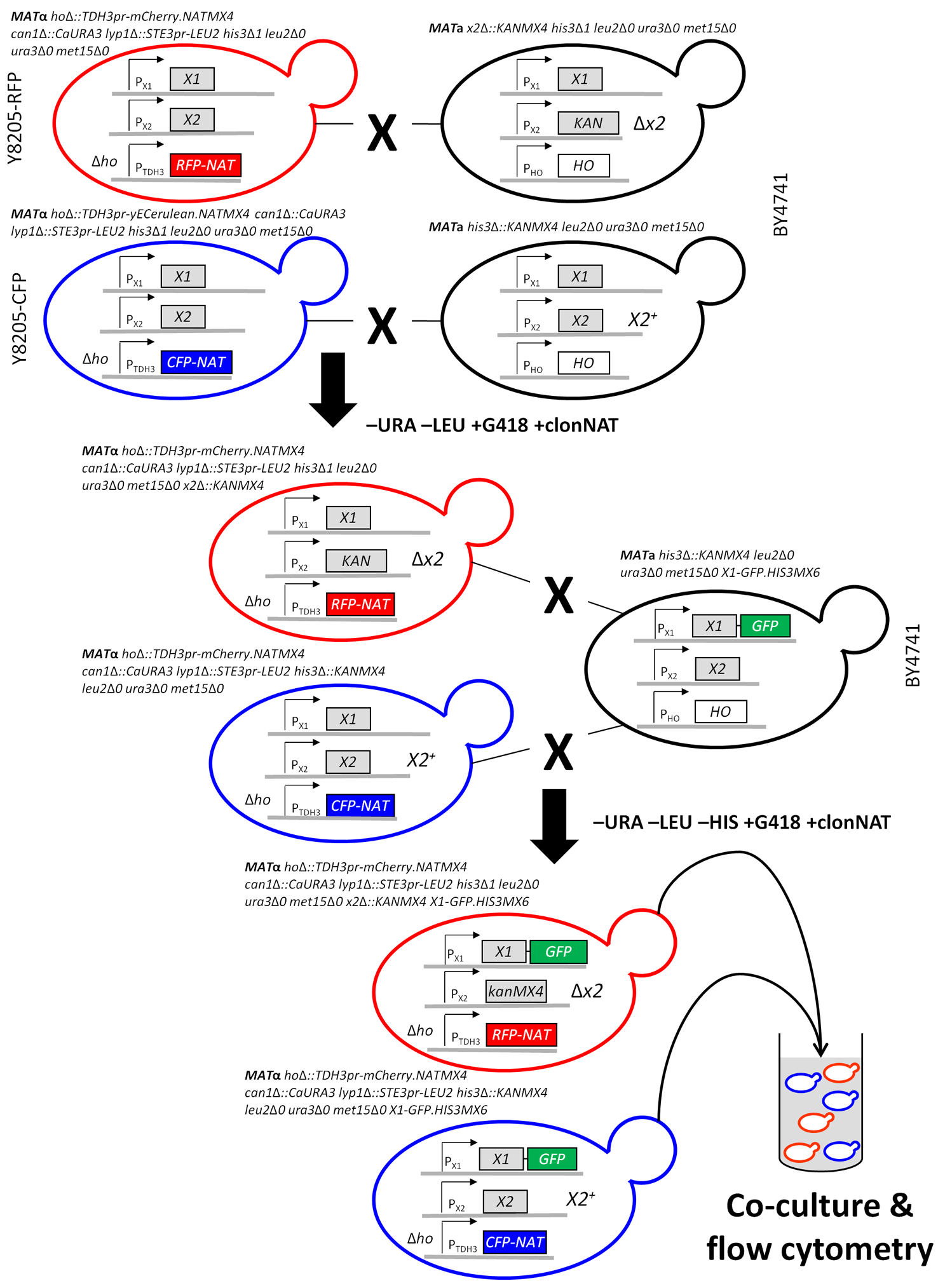

Supplement: Figure S1 — Schematic of library construction. Yeast strain libraries were generated as described in Materials and Methods. In a first SGA round, libraries of mCherry- or Cerulean-tagged deletion or wild-type strains were generated. In a second SGA round, these arrays were combined with strains from the GFP library, generating the X1-GFP Δx2 and X1-GFP X2 libraries. Black solid cross/arrows denote SGA mating, sporulation, and selection steps. (0.49 MB TIF) [file pbio.1000347.s001.tif]

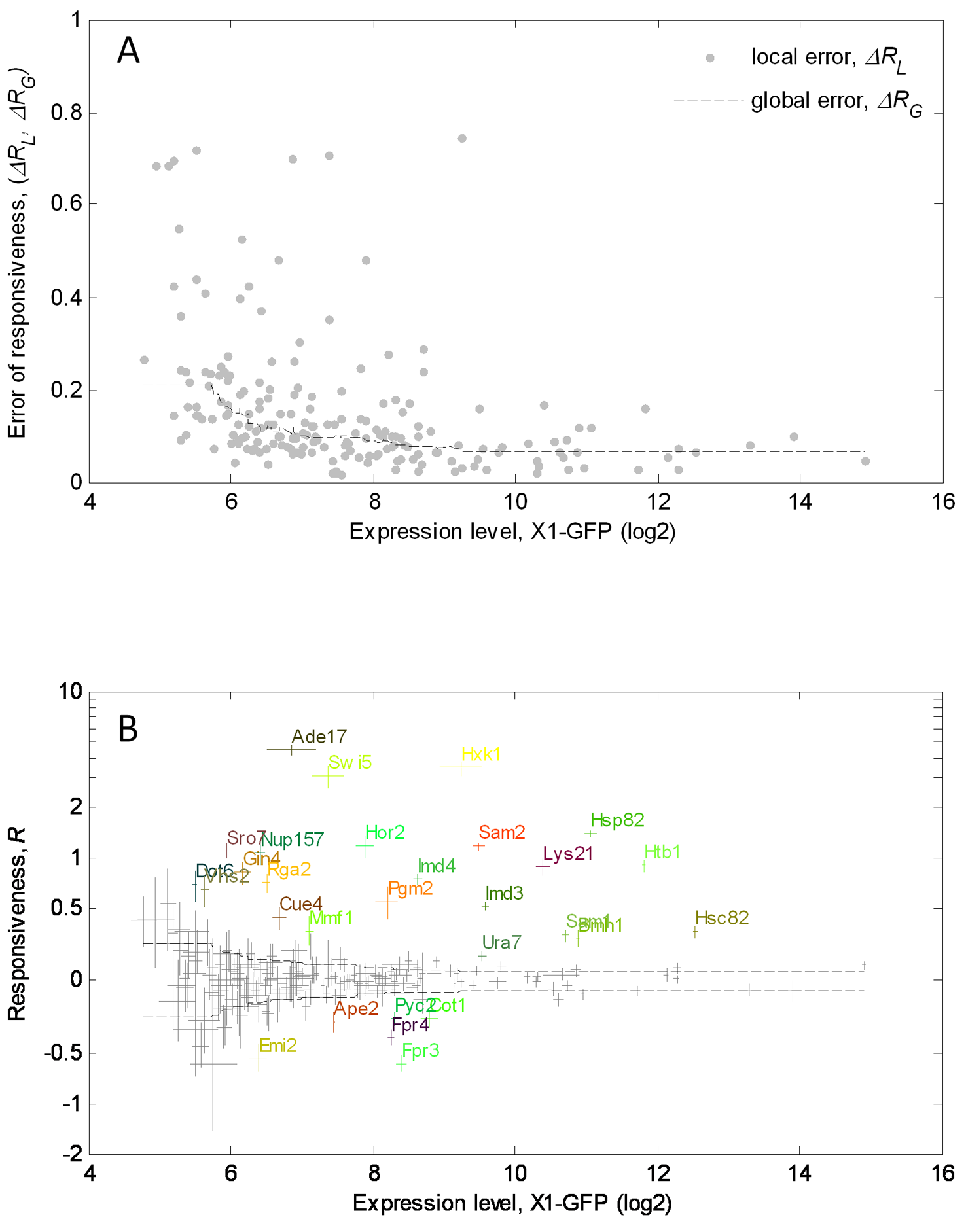

Supplement: Figure S2 — Analysis of measurement error of paralog-responsiveness. (A) Responsiveness of each gene was measured in multiple replicates representing four independently constructed strains (two of each CFP/RFP dye-swap variant), assayed in two independent replicates of the measurement procedure on different days (eight replicates total). Responsiveness R of each gene X1 was evaluated independently for each of its eight replicates as R = log2(GΔx2/GWT), where GWT, GΔx2 are the 5% truncated mean expression level of X1-GFP in the wild-type and in the Δx2 backgrounds, respectively. For each gene, the standard deviation of R in all its replicate measurements defines its “local error” ΔRL (grey dots). The global error ΔRG is then defined as the average of ΔRL over a sliding window of expression levels (dashed line, Materials and Methods). The total error for each gene ΔRT is defined by (ΔRT)2 = (ΔRL)2 + (ΔRG)2. (B) Responsiveness of each gene is plotted as a function of its wild-type expression level. Vertical error bars represent ΔRL. Dashed line indicates 2ΔRG. Significant genes have total error R/ΔRT >2 (colored names). (0.30 MB TIF) [file pbio.1000347.s002.tif]

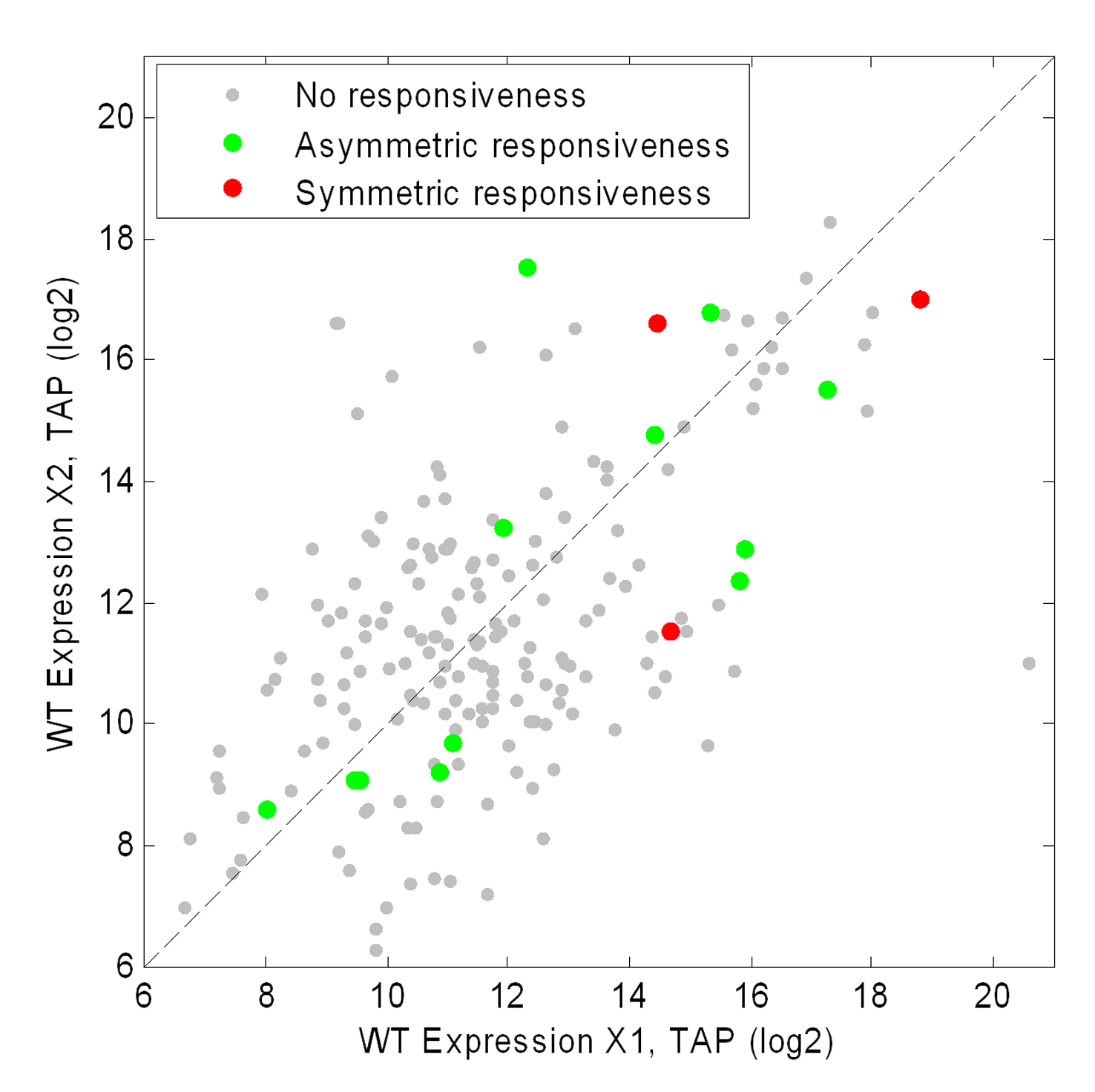

Supplement: Figure S3 — Responsiveness can be asymmetric and a property of either the low or higher or high expressed protein. Wild-type protein expression levels as determined by Western blot of TAP-tagged proteins [67] are compared for each paralogous pair. Red dots represent pairs where both paralogs are responsive, green dot where one of the two paralogs is responsive, and grey dots where neither of the paralogs are responsive. When one pair is responsive, the responsive protein expression level is plotted on the x-axis. As responsiveness is limited to cases where we measured GFP expression, a subset of the grey dots could be green (asymmetrically responsive gene pairs) or red (symmetrically responsive), and a subset of the green dots could be red (symmetrically responsive). (0.21 MB TIF) [file pbio.1000347.s003.tif]

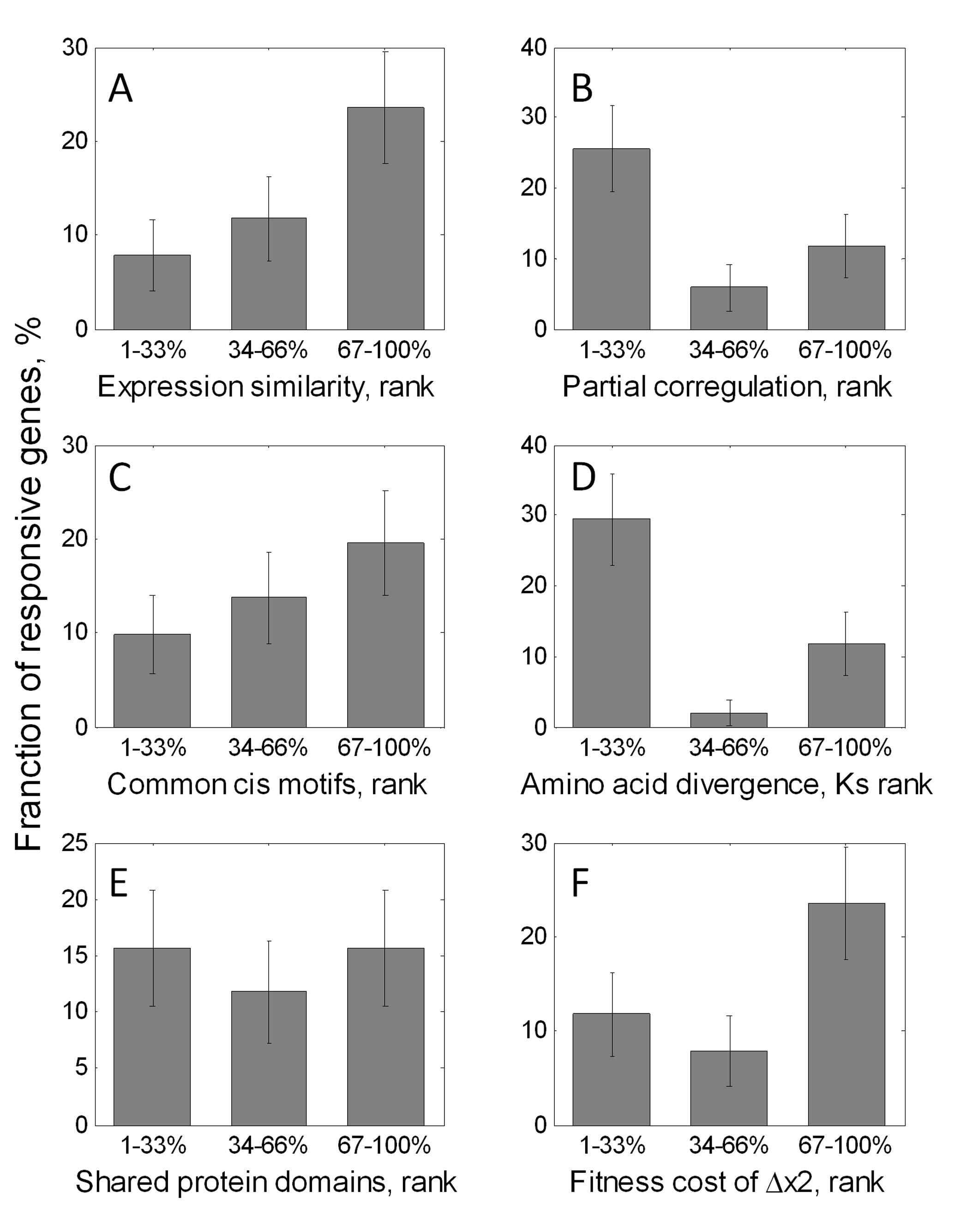

Supplement: Figure S4 — Responsiveness correlates with conservation of expression profiles, regulatory motifs, amino acid sequence, and fitness cost of paralog deletion. (A–F) Fraction of responsive genes as a function of (A) mean expression similarity, (B) partial coregulation, (C) fraction of common cis-regulatory motifs, (D) Ks rate of amino acid divergence, (E) number of shared protein domains, and (F) fitness cost upon deletion of the X2 paralog, as downloaded from Kafri et al. (http://longitude.weizmann.ac.il/BackUpCircuits/) [26]. The relevant dataset was ranked and split into three groups of equal data size. Error bars indicate standard error of the mean. (0.48 MB TIF) [file pbio.1000347.s004.tif]

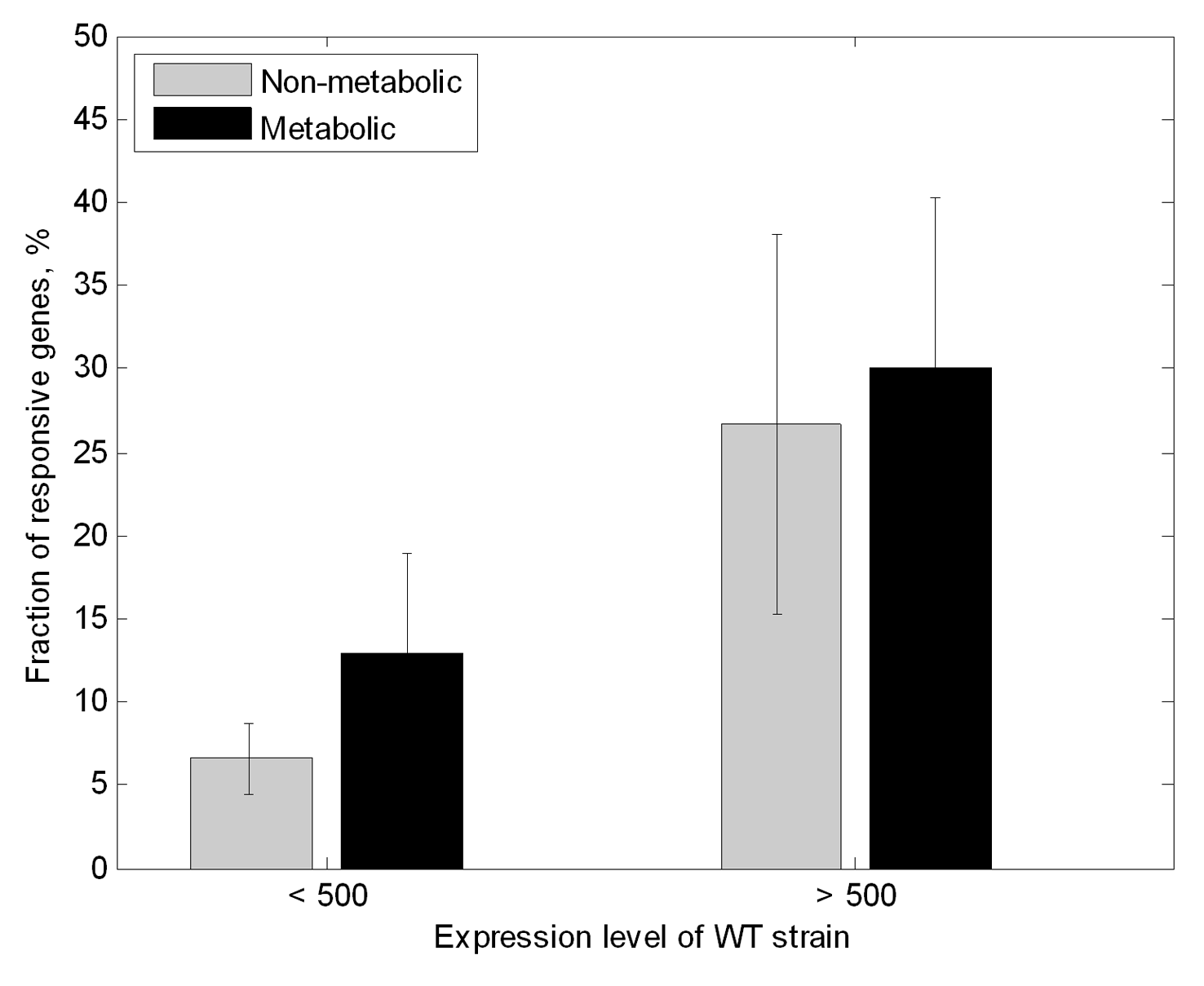

Supplement: Figure S5 — Paralog-responsiveness is enriched in highly expressed proteins. Fraction of responsive genes are shown for protein fusions with low (GWT >500) and high (GWT >500) expression levels, separated into metabolic (black) and nonmetabolic (grey) genes. Error bars represent binomial standard error of the mean. Paralog responsiveness is enriched in highly expressed proteins (p = 0.01) and slightly in metabolism (p = 0.037). Enrichment in highly expressed proteins is significant even when accounting for enrichment in metabolism and for the correlation of metabolism with high expression (p = 0.007, logit regression). (0.11 MB TIF) [file pbio.1000347.s005.tif]

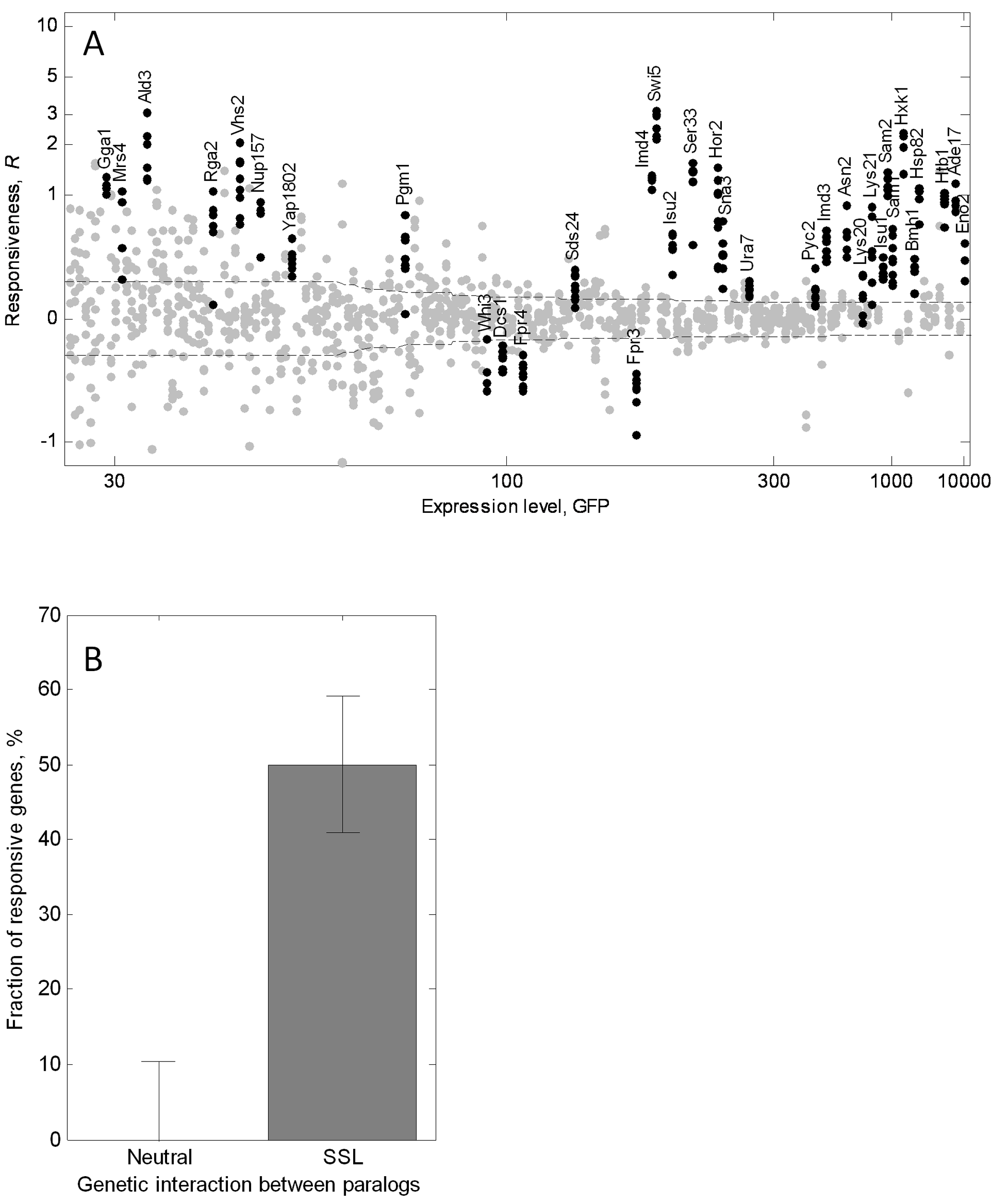

Supplement: Figure S6 — Paralog responsiveness in minimal media is strongly correlated with synthetic sick and lethal interactions. (A) Shown are all the measurements for paralog responsiveness, R, in minimal media, including replicate experiments for each gene (multiple dots in each column). Significantly responding genes are indicated (R/ΔRT >2, black dots). Genes are organized by their wild-type expression level as indicated on the x axis (see Figure 2B, for the equivalent presentation of responsiveness in rich medium). (B) Fraction of paralog-responding genes in minimal media are shown for gene pairs with no genetic interaction (neutral, n = 27) and for synthetic lethal or sick interactions (SSL, n = 16) in these conditions. SSL interactions are defined as ε = fx1x2 − fx1 fx2 <−0.2, where ε is the epistasis and fx1x2, fx1, and fx2 are the fitness values for the double and single knockouts grown in minimal medium (fitness data taken from DeLuna et al. [21]). Error bars reflect binomial standard error of the mean. All paralog-responsive genes are also synthetic lethal or synthetic sick with its paralog. (0.43 MB TIF) [file pbio.1000347.s006.tif]

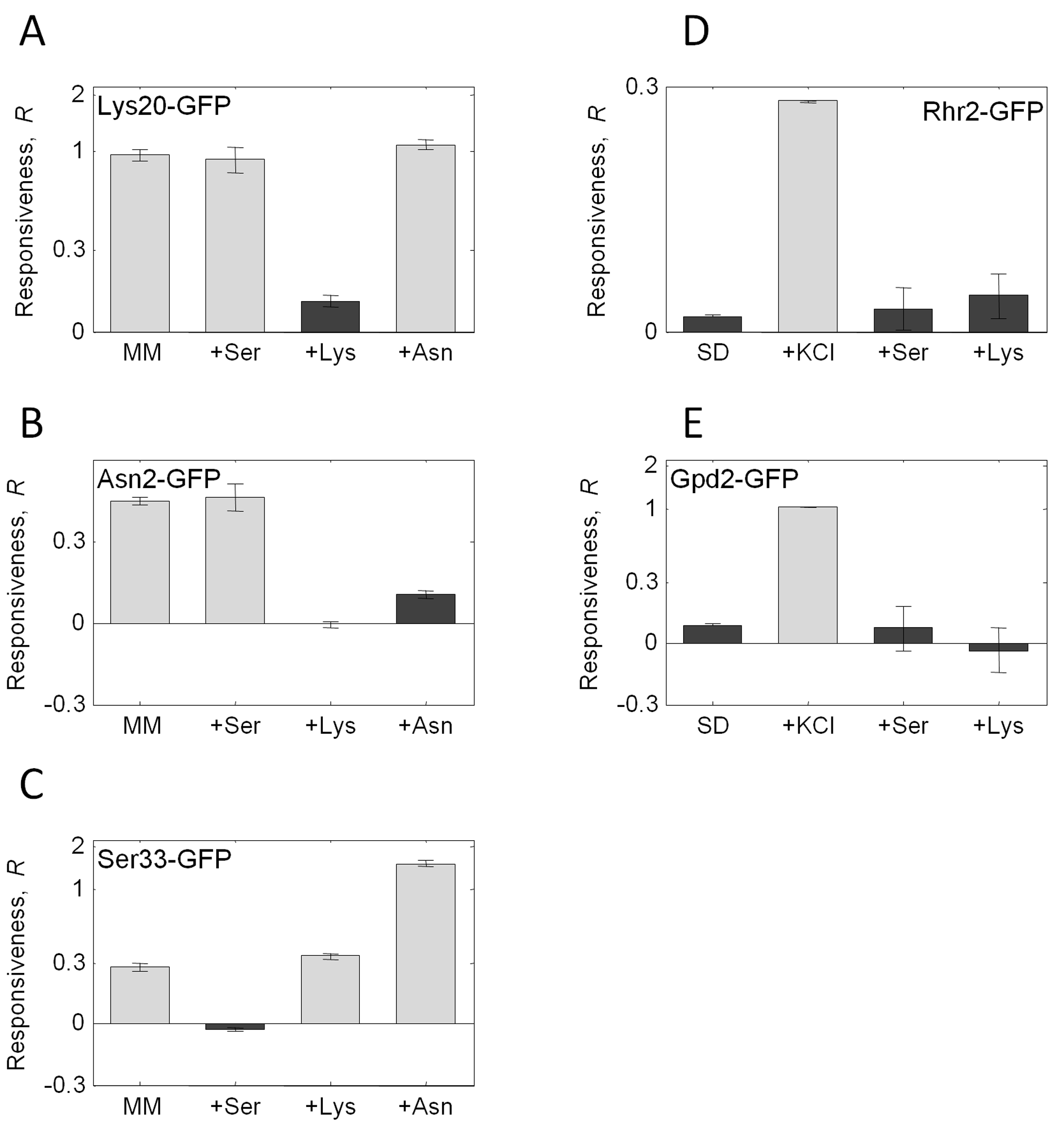

Supplement: Figure S7 — Paralog responsiveness is specific to the conditions in which the gene function is needed. (A–E) Responsiveness, R, of the focal gene in the needed (light-gray bars) or unneeded (dark-gray bars) environment: MM, minimal medium; SD, synthetic complete dextrose medium; SC+EtOH, synthetic complete ethanol medium, +Ser, minimal medium plus serine; +Lys, minimal medium plus lysine; and +Asn, minimal medium plus asparagine. The protein fusions are (A) Lys20-GFP, (B) Asn2-GFP, (C) Ser33-GFP, (D) Rhr2-GFP, and (E) Gpd2-GFP. Responsiveness of these genes is greatly reduced when cells are grown in conditions in which the genes are not needed. An exception is Asn2, which stops responding not only in the presence of asparagine, but also in the presence of lysine. R reflects the median responsiveness value of three to 11 replicate experiments. Error bars indicate standard error of the mean. (0.30 MB TIF) [file pbio.1000347.s007.tif]

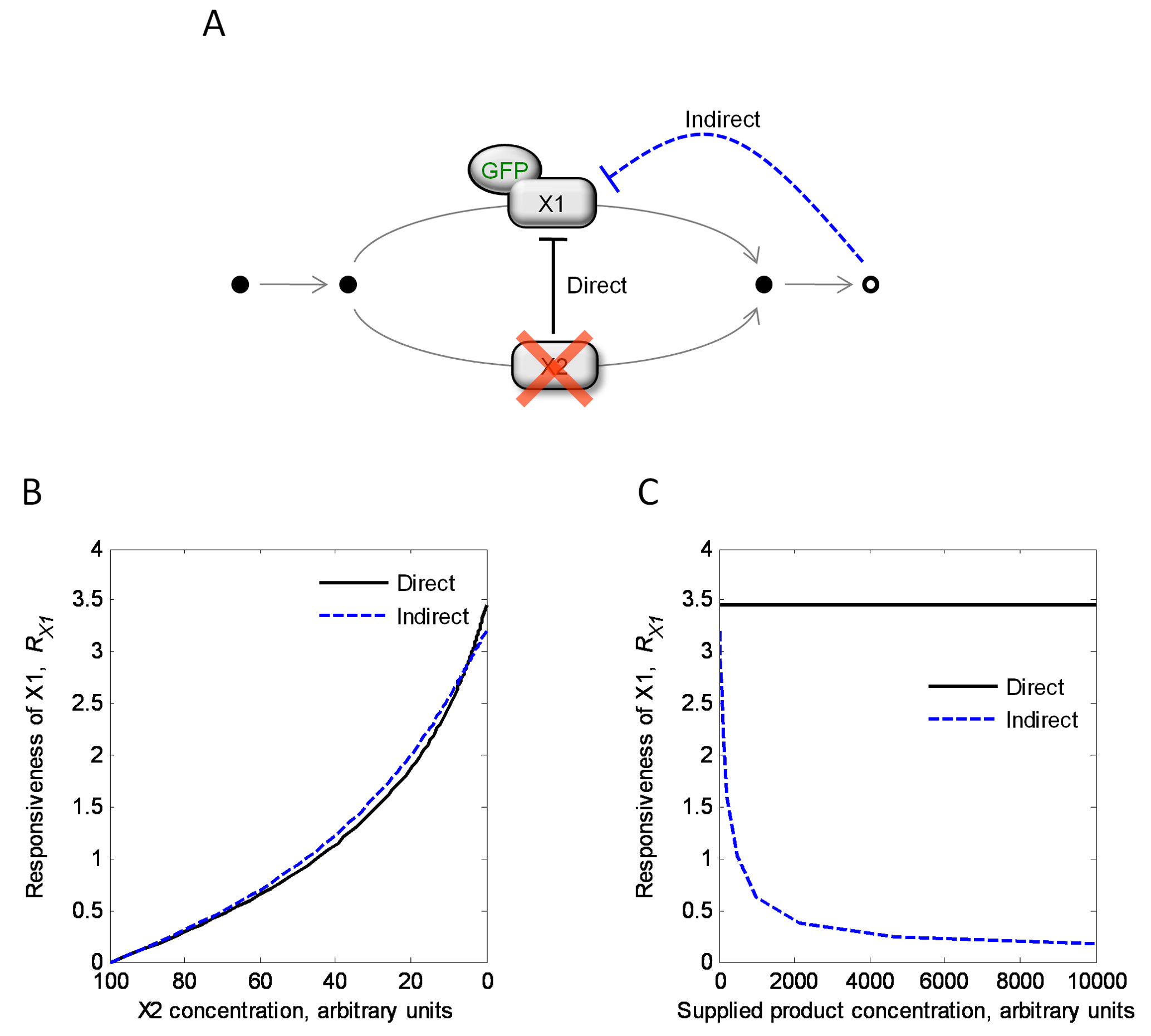

Supplement: Figure S8 — A model for direct and indirect paralog responsiveness. (A) A simple metabolic pathway showing enzymatic reactions (grey arrows) between metabolites (black circles). A gene X1 (tagged with GFP) may respond to deletion of its paralog X2 by two conceptual ways: (1) directly, in response to the absence of the paralogous protein (black solid inhibitory line), or (2) indirectly, in response to the absence of the function of the gene, for example through inhibition by the pathway end product (blue dashed inhibitory line). Mathematical models for gene expression in these two schemes were created (Text S2). (B) In an environment with a fixed amount of the end product, direct and indirect regulation of X1 in response to change in concentration of X2 are almost indistinguishable. (C) Responsiveness of X1 to deletion of X2 (X2 = 0) in the two models can be distinguished by supplying the pathway product. (0.29 MB TIF) [file pbio.1000347.s008.tif]

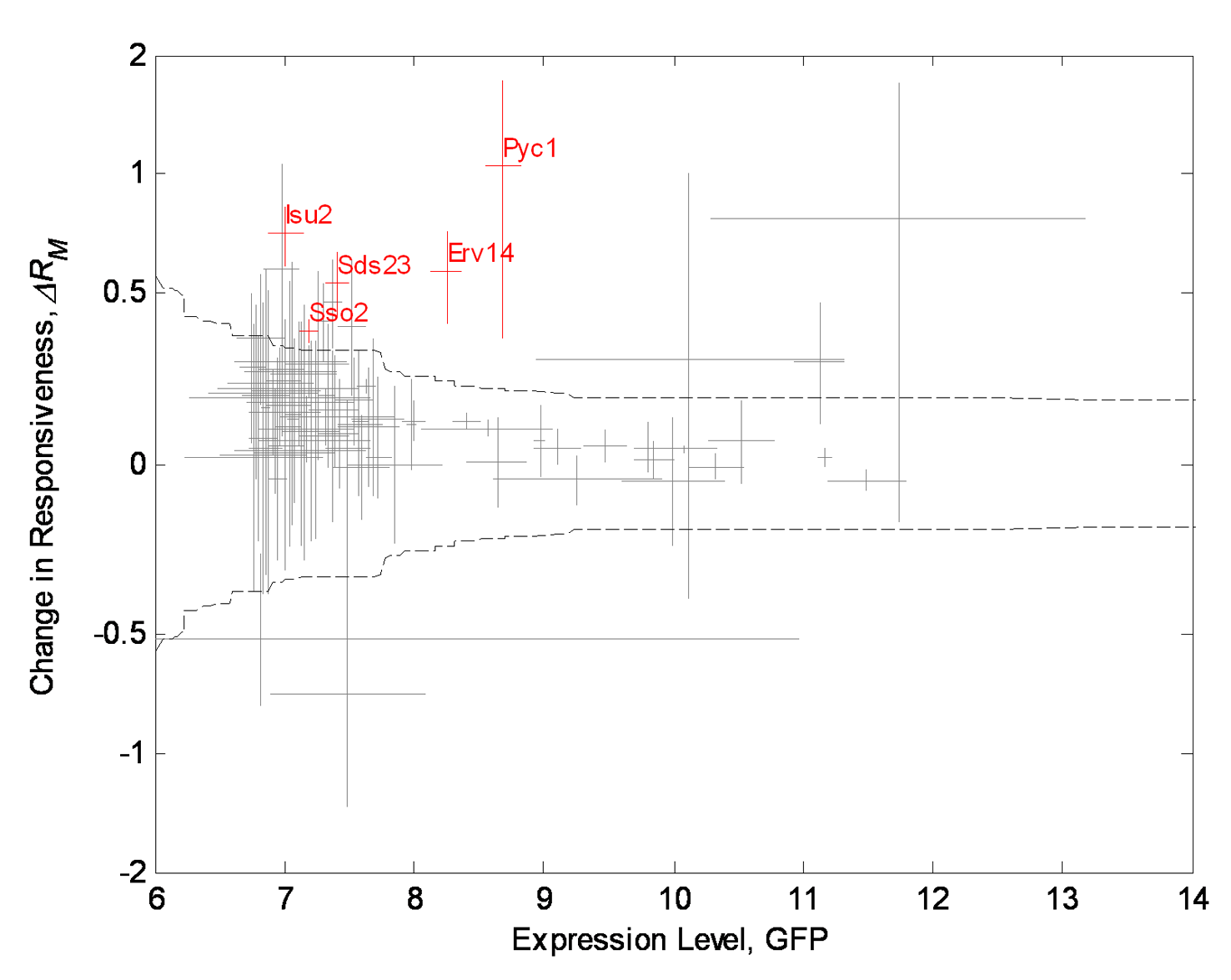

Supplement: Figure S9 — Methionine concentration has a minimal effect on the measurement of responsiveness. Responsiveness was measured in duplicate at two methionine concentrations, 25 mg/l and 100 mg/l, for one fourth of the library. The difference in responsiveness between these two environments, ΔRM (R for growth in 100 mg/l methionine minus R for growth in 25 mg/l methionine) is plotted as a function of average log2 expression of the 25 mg/l methionine-grown strain. Local and global errors are indicated (RL, error bars; RG, dashed line; Materials and Methods). Gray dots do not change significantly between conditions; five proteins Isu2, Sds23, Sso2, and Pyc1 have significant changes in responsive between the conditions. (0.15 MB TIF) [file pbio.1000347.s009.tif]

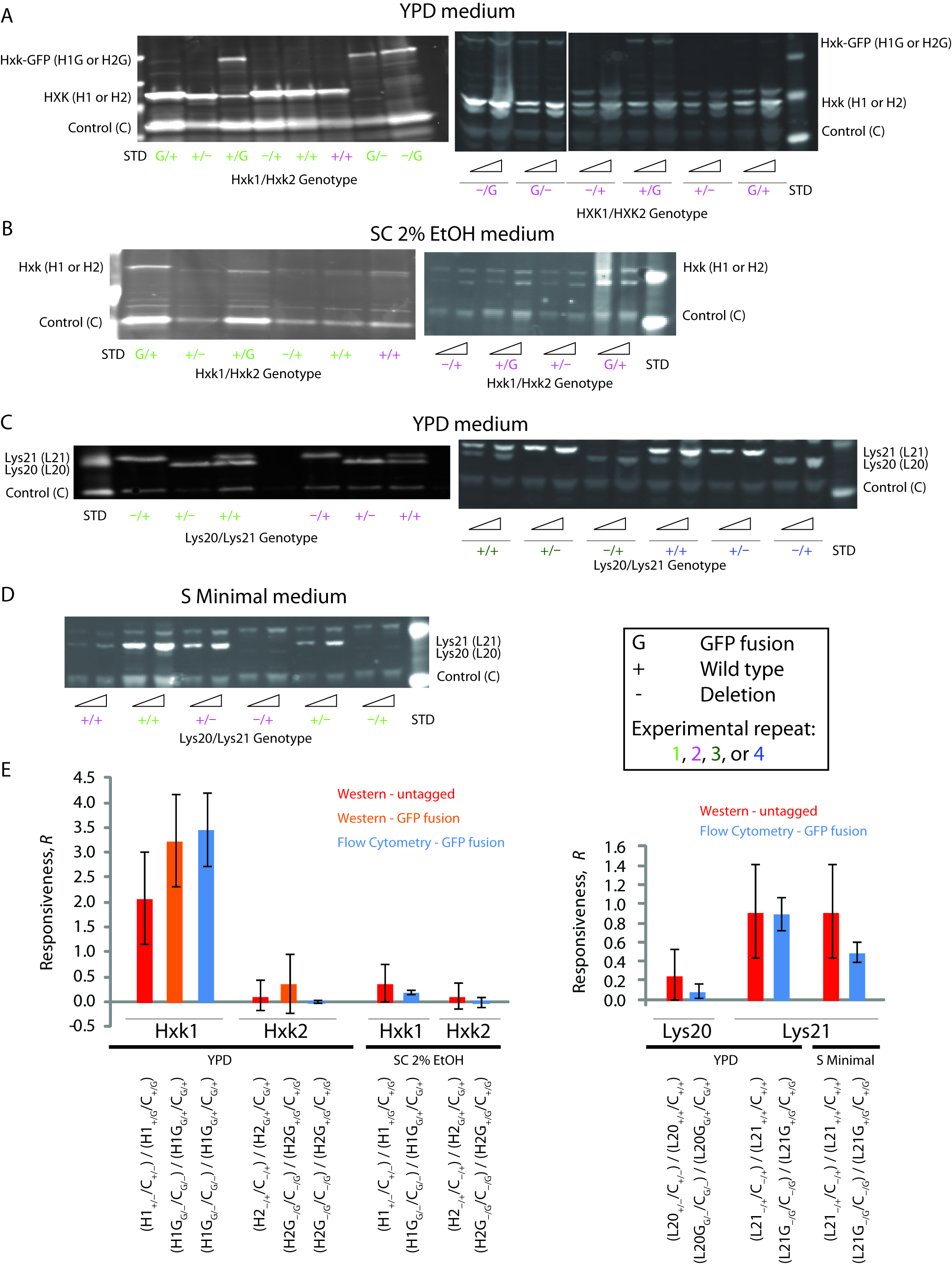

Supplement: Figure S10 — Western blots of untagged proteins confirm responsiveness of GFP-fusion proteins. (A–D) Hxk1 (H1), Hxk2 (H2), Hxk1-GFP (H1G), Hxk2-GFP (H2G), Lys20 (L20), Lys21 (L21), and a control protein (Act1, Lys20/21, or HXK1,2 [C, control]; see Materials and Methods) were detected by quantitative Western blot. The genotype of each strain used is listed beneath each lane (STD, protein standard; G, GFP fusion; +, wild-type untagged protein; –, deletion). Titration triangles indicate a 2-fold dilution of the sample. Lys20 and Lys21 could be resolved on a SDS-PAGE gel, but Hxk1 and Hxk2 could not. To resolve Hxk1 and Hxk2, each was GFP tagged to alter its mobility from the untagged protein being queried. Samples were grown in (A and C) YPD, (B) SC + 2% EtOH, and (C) minimal medium. (D) All the measurements were quantitated with a fluorescent secondary using the Odyssey software (Materials and Methods), and the responsiveness R was calculated as the ratio of its level in the mutant and the wild type corrected for the loading controls (specific formula indicated below each bar). The error bars represent the standard deviation of the replicate measurements. Responsiveness is not significantly altered by tagging or method of quantitation (Western versus flow cytometry). (1.72 MB TIF) [file pbio.1000347.s010.tif]
