# Supplementary material for: Need-Based Up-Regulation of Protein Levels in Response to Deletion of Their Duplicate Genes
Source: PLoS Biol. 2010 Mar 30;8(3):e1000347. doi: 10.1371/journal.pbio.1000347 (PMC2846854; doi:10.1371/journal.pbio.1000347)
Supplement: Text S1 — Regulatory mechanisms underlying responsiveness under different environmental conditions. (0.05 MB DOC) [file pbio.1000347.s014.doc]

### Text S1. Regulatory mechanisms underlying responsiveness under different environmental conditions

**Responsiveness of amino acid biosynthesis enzymes.** We confirmed that responsiveness in minimal medium disappears when the specific amino acid is provided, that is, in an environment in which the gene is not needed (Figure 5A-F). Such conditional need is confirmed by the fact that double mutants of *LYS20*-*LYS21*[1], *ASN1*-*ASN2*[2], and *SER3*-*SER33*[3], and are synthetic lethal in minimal medium, but viable if lysine, serine, or asparagine, respectively, is added to the growth medium.

The *LYS20* and *LYS21* encode two homocitrate synthase isoenzymes—the first committed step in the lysine biosynthesis pathway. These proteins respond to deletion of their paralogs in minimal medium lacking lysine, but stop responding if lysine is added (Figure 5A,B). Although gene expression decreases in the wild-type, it should be noted that Lys20 is still present at high levels in the presence of lysine (Figure 5A). In other words, the loss of responsiveness is not simply due to a complete loss of gene expression after addition of lysine. The activation ability of the main activator of the pathway, Lys14, is strongly enhanced when lysine is lacking in the medium[4]. Thus, it is most likely that lysine levels in the single knockouts drop, which in turn activates expression of the intact paralog through Lys14.

Similarly, Asn2 is another biosynthetic enzyme that moderates response to deletion of its paralog when the amino acid, asparagine, is added to the medium (Figure 5B). Asn1 and Asn2 are two asparagine synthetases that carry out the first and sole step of asparagine synthesis in yeast; both genes are subject to general amino acid regulation through the action of Gcn4p[2]. We observe that Asn2 is the secondary isoform present in minimal medium, but it is up-regulated when the *ASN1*-encoded isoform is missing. This responsiveness disappears if asparagine is present in the medium. We note however that protein levels of Asn2p increase in asparagine; in contrast to Asn1 decreases under this condition (data not shown). Asn2 falls into an interesting category of responding genes that seem to play a secondary role in a particular condition but take over when their paralogs are missing. Here, an active response underlies synthetic lethality of the duplicate genes, namely the gene is only required if the paralog is missing.

*SER33* participates in the first step of the serine biosynthesis pathway, and together with its paralog *SER3*, encodes two yeast 3-phosphoglycerate dehydrogenases involved in serine and glycine synthesis. These enzymes have also been shown to play an important role in yeast redox metabolism[3]. Ser33 is the main isoform expressed in exponential growth and is less sensitive to Gcn4 amino acid biosynthesis control[3]. We find that response of Ser33 to deletion of Ser3 disappears when serine is added to the minimal growth medium (Fig 5C). Given that the Ser33 is not affected by serine availability and that Ser3 does not seem to contribute substantially to serine synthesis in the wild-type, it is unlikely that up-regulation of Ser33 in the Δ*ser3* strain is mediated through end-product availability, as in the case of the lysine biosynthesis pathway.

**Responsiveness of hexokinase.** The Hxk1 paralog responds to deletion of *HXK2* in either rich or minimal glucose media (Figure 4). We predicted that the function this first irreversible step of glycolisis will not be needed when cells are grown under a non-fermentable carbon source. Indeed, we find that the strong responsiveness of Hxk1 in minimal glucose medium is completely abolished when cells grow on ethanol as a source of carbon (Figure 5D), that is, responsiveness disappears when the gene’s function is not needed. The Hxk1 appears as an interesting case of direct regulation of responsiveness through its paralog, Hxk2. Both absence of glucose or absence of Hxk2 results in Hxk1 upregulation, in consistence with previous observations showing that nuclear Hxk2 is involved in repression of *HXK1* and expression of its own gene, [*HXK2*](http://www.yeastgenome.org/cgi-bin/locus.fpl?dbid=S000003222)[5,6]. Repression of *HXK1* through Hxk2 depends on Mig1 and glucose, and thus disappears in yeast cells grown on ethanol.

**Responsiveness of glycerol biosynthesis enzymes.** We asked if we could find proteins that do not respond in either rich or minimal medium and indentify conditions that will induce a response. To this end, we focused our attention to proteins that are present at equal levels in the wild type and in the paralog-deletion strains (Figure 4). In particular, we analyzed two non-responding proteins in the glycerol biosynthesis pathway, which are known to play a role in protection against osmotic stress. Strikingly, Rhr2 is strongly upregulated in the paralog Δhor2 strain grown in the presence of 0.5 M KCl, but shows no response in minimal medium without KCl (Figure 5E). Similarly, We observe that Gpd2 is not up-regulated by osmotic stress in the wild type, consistent with previous observations[7], but interestingly it does respond to osmotic stress when Gpd1 is missing (Figure 4F). Such up-regulation is not observed in the absence of KCl; responsiveness of Gpd2 is specific to the condition in which it is needed. Gpd2 has been found to play a secondary role in protection to osmotic stress[7], but interestingly takes over this role when its paralog is missing.

**References for Text S1**

1. Quezada H, Aranda C, DeLuna A, Hernandez H, Calcagno ML, et al. (2008) Specialization of the paralogue LYS21 determines lysine biosynthesis under respiratory metabolism in Saccharomyces cerevisiae. Microbiology 154: 1656-1667.

2. Dang VD, Valens M, Bolotin-Fukuhara M, Daignan-Fornier B (1996) Cloning of the ASN1 and ASN2 genes encoding asparagine synthetases in Saccharomyces cerevisiae: differential regulation by the CCAAT-box-binding factor. Molecular Microbiology 22.

3. Albers E, Laize V, Blomberg A, Hohmann S, Gustafsson L (2003) Ser3p (Yer081wp) and Ser33p (Yil074cp) Are Phosphoglycerate Dehydrogenases in Saccharomyces cerevisiae. J Biol Chem 278: 10264-10272.

4. Feller A, Dubois E, Ramos F, Pierard A (1994) Repression of the genes for lysine biosynthesis in Saccharomyces cerevisiae is caused by limitation of Lys14-dependent transcriptional activation. Mol Cell Biol 14: 6411-6418.

5. Rodríguez A, De La Cera T, Herrero P, Moreno F (2001) The hexokinase 2 protein regulates the expression of the GLK1, HXK1 and HXK2 genes of Saccharomyces cerevisiae. Biochem J 355: 625-631.

6. Ahuatzi D, Herrero P, de la Cera T, Moreno F (2004) The Glucose-regulated Nuclear Localization of Hexokinase 2 in Saccharomyces cerevisiae Is Mig1-dependent. J Biol Chem 279: 14440-14446.

7. Ansell R, Granath K, Hohmann S, Thevelein JM, Adler L (1997) The two isoenzymes for yeast NAD(+)-dependent glycerol 3-phosphate dehydrogenase encoded by GPD1 and GPD2 have distinct roles in osmoadaptation and redox regulation. Embo Journal 16: 2179-2187.
