# Supplementary material for: Need-Based Up-Regulation of Protein Levels in Response to Deletion of Their Duplicate Genes
Source: PLoS Biol. 2010 Mar 30;8(3):e1000347. doi: 10.1371/journal.pbio.1000347 (PMC2846854; doi:10.1371/journal.pbio.1000347)
Supplement: Text S2 — Model for direct and indirect responsiveness. (0.10 MB DOC) [file pbio.1000347.s015.doc]

### Text S1. Model for direct and indirect responsiveness

To better understand the potential differences between indirect and direct paralog-responsiveness, we analyzed two simple mathematical models of linear metabolic pathways with either direct or indirect regulation of protein abundance. We used these models to help determine what types of experiments could separate between indirect and direct regulation.

The following two systems of equations follow the time evolution of our regulated paralog, *X1*, and the end-product of the pathway, *P* (Figure S8A). Production of *X1* depends on *P* in the indirect model (Eq. 1) and X2 in direct model (Eq. 2). In both the indirect and direct mode, product formation, *P*, depends on the concentration of paralogs *X1 and X2* (Eq3).

Indirect regulation of *X1*:

$\frac{dX_{1}}{dt}=\alpha_{X}(\frac{K_{P}}{K_{P}+P})-\beta X_{1}$ (1)

Direct regulation of *X1*:

$\frac{dX_{1}}{dt}=\alpha_{X}(\frac{K_{X}}{K_{X}+X_{2}})-\beta X_{1}$ (2)

Production of product:

$\frac{dP}{dt}=\alpha_{P}(X_{1}+X_{2})-\beta P$ (3)

We assume that the indirect or direct inhibitory relationship follows a Michaelis-Menten relationship with a Michaelis constant of *K_p_* or *K_x_* respectively. All components are diluted by growth, β.

We numerically modeled the system using MATLAB (MathWorks). We tested a range of values for each constant, the parameters used in figure S8B and C are given in the table below. Deleting one paralog and monitoring the concentration of the other paralog is insufficient to distinguish between direct and indirect feedback (Figure S8B). While our experiments represented in Figure 2 help to highlight proteins that are responsive, these experiments cannot help to discriminate mechanism.

We next expanded our models to include a situation where we add large amounts of exogenous product to the system. We do this by adding a term,$Ts(\frac{Ps}{Ps+K_{PS}})$, to the differential equations controlling the end-product level (Eq. 3 becomes Eq. 4). This term controls the rate at which externally supplied product, *Ps*, can influence the concentration of *P*. *Ts* is the maximal rate at which *Ps* can be imported by the cell and *K_PS_* is the Michaelis constant for the uptake.

Indirect regulation in the presence of varying levels of added product:

$\frac{dP}{dt}=\alpha_{P}(X_{1}+X_{2})-\beta P+Ts(\frac{Ps}{Ps+K_{PS}})$ (4)

Where

$$\alpha_{P}\equiv\alpha(\frac{K_{s}}{K_{s}+S})$$

α is the Vmax of the enzymes. *K_s_* is the Michaelis constant for the substrate. *S* is the substrate concentration.

**Constants, description and values for the models of Figure S8.**

| **Symbol** | **Definition** | **Units** | **Values** |
| --- | --- | --- | --- |
| *α_x_* | Maximal *X1* production rate | uM/min | 10 |
| *K_x_* | Michaelis constant for inhibition of *X1* production by *X2* | uM | 10 |
| *β* | Growth rate | min^-1^ | 1/90 |
| *α_p_* | Maximal rate of production of *P* by *X1* and *X2* | uM/min | 10 |
| *K_P_* | Michaelis constant for inhibition of *X1* production by *P* | uM | 10 |
| *T_s_* | Maximal rate at which externally supplied *P* enters the cells | mM/min | 1 |
| *K_PS_* | Michaelis constant for import of externally supplied *P* | mM | 1 |

We numerically modeled these systems for a range of parameters. A representative behavior is shown in Figure S8C with the parameter values listed in the table. In contrast to the situation where only the paralog level is changed, when product is supplied, the two mechanisms can be distinguished. Direct regulation is not affected by product addition while indirect regulation is affected. This experiment would not be able to separate whether indirect regulation was occurring alone or in combination with direct regulation. This experiment can solely rule out indirect regulation. Therefore the experiments we perform associated with Figure 5 can determine whether indirect regulation is present.
